# Neuropharmacological Evidence Implicating Drug-Induced Glutamate Receptor Dysfunction in Affective and Cognitive Sequelae of Subchronic Methamphetamine Self-Administration in Mice

**DOI:** 10.3390/ijms25031928

**Published:** 2024-02-05

**Authors:** Christopher J. E. Denning, Lauren E. Madory, Jessica N. Herbert, Ryan A. Cabrera, Karen K. Szumlinski

**Affiliations:** 1 Department of Psychological and Brain Sciences, University of California Santa Barbara, Santa Barbara, CA 93106, USA; christopherdenning@ucsb.edu (C.J.E.D.); madory@ucsb.edu (L.E.M.); jessicanicoleherbert@gmail.com (J.N.H.); ryanacabrera@ucsb.edu (R.A.C.); 2 Department of Molecular, Cellular and Developmental Biology, University of California Santa Barbara, Santa Barbara, CA 93106, USA

**Keywords:** NMDA receptor, Group 1 mGluR, anxiety, sex differences, spatial learning, working memory, reversal learning

## Abstract

Methamphetamine (MA) is a highly addictive drug, and MA use disorder is often comorbid with anxiety and cognitive impairment. These comorbid conditions are theorized to reflect glutamate-related neurotoxicity within the frontal cortical regions. However, our prior studies of MA-sensitized mice indicate that subchronic, behaviorally non-contingent MA treatment is sufficient to dysregulate glutamate transmission in mouse brain. Here, we extend this prior work to a mouse model of high-dose oral MA self-administration (0.8, 1.6, or 3.2 g/L; 1 h sessions × 7 days) and show that while female C57BL/6J mice consumed more MA than males, MA-experienced mice of both sexes exhibited some signs of anxiety-like behavior in a behavioral test battery, although not all effects were concentration-dependent. No MA effects were detected for our measures of visually cued spatial navigation, spatial learning, or memory in the Morris water maze; however, females with a history of 3.2 g/L MA exhibited reversal-learning deficits in this task, and mice with a history of 1.6 g/L MA committed more working-memory incorrect errors and relied upon a non-spatial navigation strategy during the radial-arm maze testing. Relative to naïve controls, MA-experienced mice exhibited several changes in the expression of certain glutamate receptor-related proteins and their downstream effectors within the ventral and dorsal areas of the prefrontal cortex, the hippocampus, and the amygdala, many of which were sex-selective. Systemic pretreatment with the mGlu1-negative allosteric modulator JNJ 162596858 reversed the anxiety-like behavior expressed by MA-experienced mice in the marble-burying test, while systemic pretreatment with NMDA or the NMDA antagonist MK-801 bi-directionally affected the MA-induced reversal-learning deficit. Taken together, these data indicate that a relatively brief history of oral MA is sufficient to induce some signs of anxiety-like behavior and cognitive dysfunction during early withdrawal that reflect, at least in part, MA-induced changes in the corticolimbic expression of certain glutamate receptor subtypes of potential relevance to treating symptoms of MA use disorder.

## 1. Introduction

The 2023 World Drug Report estimates that nearly 1% of the world’s population (approximately 36 million people) reported using amphetamine-type stimulants (ATSs) in 2021. This is a significant increase over the past decade [1], and in the United States alone, overdose deaths involving methamphetamine (MA) have increased more than 5-fold over the past 10 years [2]. In humans, MA induces a negative affective state that is dose-dependent and characterized by anxiety and dysphoria from within the first 24 h to a few days into drug abstinence [3]. The severity of this state is intensified when individuals engage in high-dose, binge-like patterns of MA use [4,5]. Further, MA use disorder is associated with impairment across a number of cognitive domains, including episodic and working memory [6,7,8,9,10,11]. Concerningly, the gender gap in ATS use is closing, with 45% of current ATS users identifying as women [1]. Concerningly, despite the observation that women progress along the addiction landscape faster than men [12,13] and experience more comorbid psychiatric symptoms [14], globally, only 1 in 4 women receive treatment for their ATS use disorder [1]. Thus, there is an urgent need to better understand how a history of high-dose ATS use impacts the brains of both women and men, which is of relevance not only to sex differences in the development of addiction [12,13] but also to other comorbid psychiatric conditions that can severely impact the disease prognosis and treatment [14]. 

Oral MA intake can lead to problematic drug use, although the subjective experience is different between the intravenous and nasal routes of administration [3]. Further, the oral route of MA administration is also associated with a high level of toxicity [15] due to the relatively slow rates of MA absorption and clearance that result in high blood MA levels [3,16]. Both mice (e.g., [17,18,19,20,21,22,23]) and rats (e.g., [24,25]) will consume MA orally, despite their higher rate of MA metabolism than humans [26,27]. Although the majority of oral MA self-administration studies have employed relatively low MA concentrations (from 0.02 to 0.80 g/L), we recently showed that both male and female inbred C57BL/6J (B6J) or C57BL6/NJ mice can be trained to operantly respond to unadulterated MA solutions as high as 3.2 g/L and will orally consume upwards of 30 mg/kg MA during a 1 h session [17]. Heavy MA consumption in humans is roughly 10–13 mg/kg/day for a 77 kg (170 lb) person [26,28], with binge-like MA consumption reaching from 2 to 3 times that amount [29]. Thus, our procedures provide a clinically relevant [3,15,16] and procedurally facile model with which to study the effects of a history of binge-like MA consumption on the brain and behavior of relevance to our neurobiological understanding of MA use disorder and its treatment [17]. 

To this end, the present study is the first in a series of studies that are currently on-going in our laboratory that seek to extend our current model of high-concentration oral MA self-administration in B6J mice [17]. When trained initially to respond to a 0.1 g/L MA solution, the female mice in our earlier study consumed more MA than the males, but they did not differ from the males with respect to the dose–response function for oral MA intake once operant conditioning was acquired [17]. Thus, our first goal was to determine whether or not this sex difference in the acquisition of oral MA intake [17] extended to higher MA concentrations. For this, mice were trained to self-administer 0.8, 1.6, or 3.2 g/L MA. We next sought to determine whether a relatively brief history of binge-like MA consumption (1 week) would be sufficient to induce signs of negative affect during early (24 h) withdrawal using a behavioral test battery similar to that employed in our earlier studies of the negative affective consequences of alcohol withdrawal (e.g., [30,31,32,33]). To probe the neurocognitive consequences of binge-like MA consumption, mice were then tested under a Morris water maze and radial-arm maze procedures. Decades of evidence implicate glutamate in the behavioral and neurotoxic effects of MA (cf., [34,35,36]). Based on prior work indicating that subchronic MA injection regimens and intravenous MA self-administration are sufficient to elicit changes in both pre- and postsynaptic aspects of glutamate signaling within the brain [22,35,37,38,39,40,41,42,43], the behavioral effects of our relatively brief oral MA self-administration procedures were then related to changes in the indices of glutamate signaling within the subregions of the prefrontal cortex (PFC), the amygdala, and the hippocampus by immunoblotting for glutamate receptor-related proteins and two downstream effectors: extracellular signal-regulated kinase (ERK) and calcium/calmodulin kinase IIα (CaMKII) [44,45,46,47,48]. Finally, to begin to probe the functional relevance of our observed changes in glutamate receptor expression, we conducted two small-scale behavioral pharmacological studies designed to address the role of the mGlu1 and NMDA glutamate receptors in MA-induced negative affect and impaired reversal learning, respectively. 

We predicted that B6J mice would readily consume high-concentration MA solutions, with a higher intake in females versus males. We also predicted that if the MA intake reached binge-like amounts, then a week-long self-administration history would be sufficient to elicit emotional and cognitive disturbances, coinciding with a dysregulation in the glutamate receptor-related protein expression. Although females consumed more MA than males in the initial dose–response study, no sex difference in the MA intake was apparent in the two subsequent behavioral pharmacological studies in which mice self-administered 3.2 mg/L MA only. As hypothesized, a 1-week history of high-concentration oral MA self-administration induced some signs of anxiety-like behavior and cognitive dysfunction, although most effects were not concentration-dependent. Subchronic oral MA self-administration altered the expression of several glutamate-related proteins within the prefrontal cortex, hippocampus, and amygdala, but many of these changes were sex-selective and concentration-independent. Systemic pretreatment with an mGlu1 antagonist reversed the anxiety-like behavior expressed by MA-experienced mice in the marble-burying test, while pretreatment with the NMDA antagonist mimicked and NMDA reversed the MA-induced deficit in reversal learning in the Morris water maze. These data indicate that a relatively brief history of oral MA is sufficient to induce behaviorally relevant changes in glutamate receptors in the key brain regions governing the affective and cognitive processing of relevance to the etiology and treatment of MA-disordered behavior. 

## 2. Results

### 2.1. Sex Differences in High-Concentration Oral MA Intake

**Figure 1:**
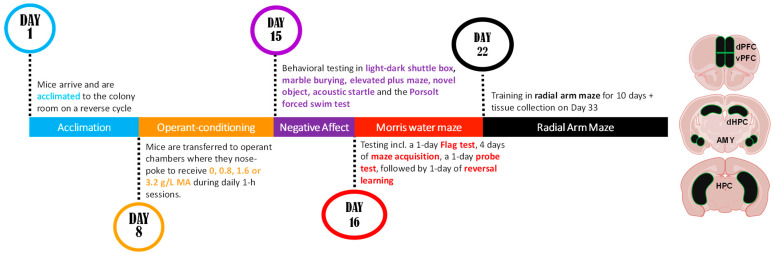
Summary of the procedural timeline employed in the dose–response study of high-concentration MA (0.8, 1.6, 3.2 g/L), during which mice were trained to nose poke for the delivery of 20 µL of their assigned MA reinforcer over the course of 7 days. The day following the end of self-administration, mice underwent a 1-day behavioral test battery to assay sensorimotor gating and negative affect. Mice were then tested under our Morris water maze and radial-arm maze procedures. Then, tissues were dissected from the ventral and dorsal aspects of the prefrontal cortex (PFC) and the hippocampus (HPC) (which were combined into one sample), as well as from the amygdala (AMY), and they were processed by immunoblotting for the protein expression of glutamate-related proteins and their downstream effectors, ERK and CaMKIIα.

To facilitate the navigation of the experimental design of our initial dose–response study, a schematic of the procedural timeline is provided in Figure 1 below.

**Figure 2:**
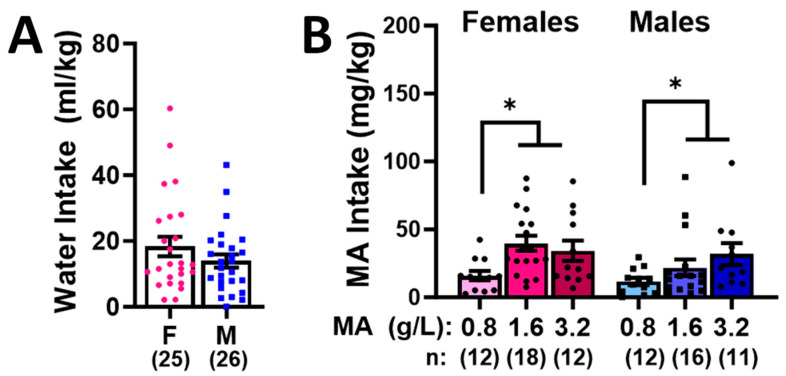
Examination of sex differences in the average total intakes of water (**A**) and different MA concentrations (**B**) during daily 1 h operant-conditioning sessions over the 7-day course of self-administration. The data represent the means ± SEMs of the numbers of mice indicated in parentheses. F denotes female; M denotes male. * *p* < 0.05 vs. 0.8 g/L MA (Tukey’s tests).

Female and male mice exhibited comparable intakes of both water (Figure 2A; *t*-test, *p* = 0.221) and MA (Figure 2B; Sex effect and interaction, *p*-values > 0.100). As expected, both male and female mice consumed more MA during the 1 h operant-conditioning session, when the 1.6 g/L and 3.2 g/L MA solutions served as the reinforcers versus the 0.8 g/L MA solution, but there was no difference in the intakes between the two higher MA concentrations (Figure 2B) [dose effect: F(2,80) = 5.742, *p* = 0.005; Tukey’s tests: 0.8 vs. 1.6: *p* = 0.01; 0.8 vs. 3.2: *p* = 0.009; 1.6 vs. 3.2, *p* = 0.951].

### 2.2. Behavioral Test Battery

The day following the last self-administration session, the mice underwent a behavioral test battery consisting of the light-dark shuttle box, marble burying, an elevated plus maze, a novel-object reactivity test, acoustic startle and the prepulse inhibition of acoustic startle (PPI), as well as a forced-swim test. The results from these assays are summarized below. 

#### 2.2.1. Marble Burying

**Figure 3:**
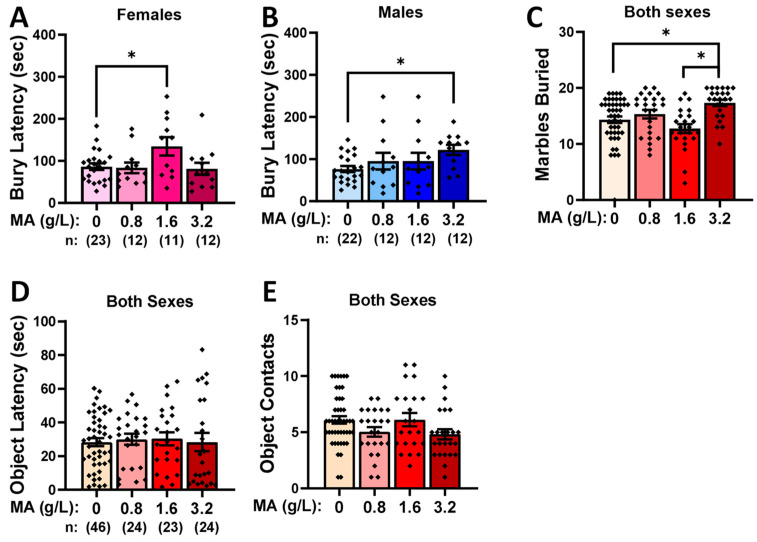
Summary of the effects of a 7-day history of oral MA (0, 0.8, 1.6, or 3.2 g/L) on neophobic-like behavior in tests of marble burying (**A**–**C**) and novel-object reactivity (**D**,**E**). The data represent the means ± SEMs of the numbers of mice indicated in parentheses. * *p* < 0.05 for specific dose comparison indicated (Tukey’s tests).

A Sex X Dose interaction was detected for the latency to begin the marble burying [F(3,113) = 4.308, *p* = 0.007]. Deconstruction of this interaction along the Sex factor revealed Dose effects for both female (Figure 3A) [F(3,57) = 3.088, *p* = 0.035] and male (Figure 3B) [F(3,55) = 2.927, *p* = 0.042] mice. In the case of females, the mice with a prior history of 1.6 g/L MA (MA-1.6) exhibited a longer bury latency than their water controls (Tukey’s tests: *p* = 0.05; other *p*-values > 0.058), while for males, the mice with a prior history of 3.2 g/L MA (MA-3.2) exhibited a longer bury latency than their water controls (Tukey’s tests: *p* = 0.039; other *p*-values > 0.101). Prior MA history also altered the number of marbles buried, but no sex difference was noted for this effect (Figure 3C) [Dose effect: F(1,116) = 6.975, *p* < 0.001; Sex effect: *p* = 0.076; interaction: *p* = 0.195]. As illustrated in Figure 3C, the MA-3.2 mice buried more marbles than both the water controls (MA-0) and the MA-1.6 mice (Tukey’s tests: *p* = 0.006 and *p* = 0.001, respectively; other *p*-values > 0.064).

#### 2.2.2. Novel-Object Reactivity

A comparable analysis of the data from the novel-object reactivity test indicated no differences for either the latency in first approaching the novel object in the center of the open field (Figure 3D; Dose X Sex ANOVA: all *p*-values > 0.078) or the number of object contacts (Figure 3E; Dose X Sex ANOVA: all *p*-values > 0.072). Taken together, these data from the marble-burying and novel-object reactivity tests fail to indicate that a consistent effect of a prior subchronic history of MA induces neophobia-like behavior during early withdrawal. 

#### 2.2.3. Elevated Plus Maze

**Figure 4:**
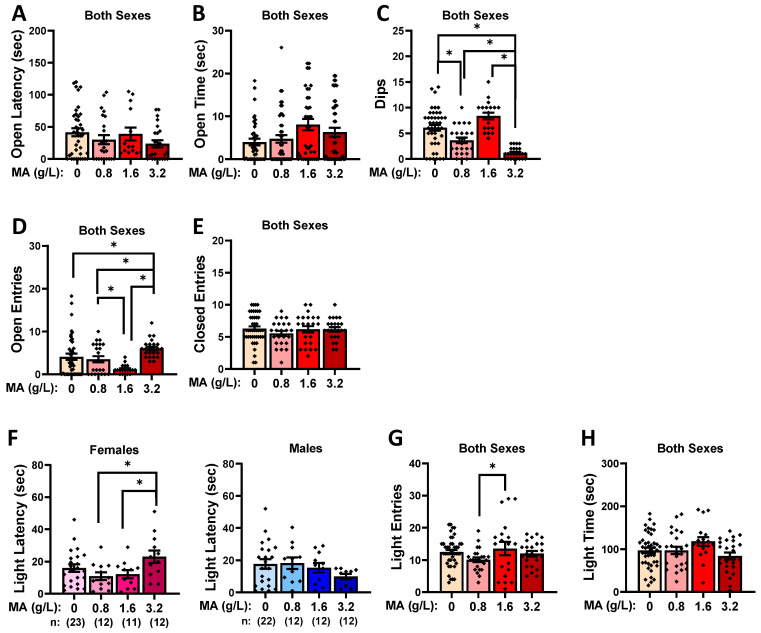
Summary of the effects of a 7-day history of oral MA (0, 0.8, 1.6, or 3.2 g/L) on agoraphobic/photophobic-like behavior in the elevated plus maze (**A**–**E**) and dark–light shuttle-box (**F**–**H**) tests. The data represent the means ± SEMs of the numbers of mice indicated in parentheses. * *p* < 0.05 for specific dose comparison indicated (Tukey’s tests).

A prior MA history did not influence the latency to first enter an open arm in the elevated plus maze (Figure 4A; Dose X Sex ANOVA: all *p*-values > 0.123) or the total time spent in the open arm (Figure 4B; Dose X Sex ANOVA: all *p*-values > 0.231), but it did affect the number of investigatory dips over the edge of the maze, irrespective of sex (Figure 4C) [Dose effect: F(1,113) = 24.290, *p* < 0.001; other *p*-values > 0.61]. As illustrated in Figure 4C, the MA-3.2 mice made the fewest dips relative to the rest of the mice (Tukey’s tests: *p*-values < 0.031), while the MA-0.08 mice also exhibited fewer dips than the water controls (Tukey’s test: *p* = 0.010; other *p*-values > 0.059). Curiously, the MA-3.2 mice entered the open arm more than all the other mice (Figure 4D) [Dose effect: F(1,114) = 18.438, *p* < 0.001; Tukey’s tests: *p*-values < 0.005], with a difference also noted between the MA-0.8 and MA-1.6 mice (Tukey’s test: *p* = 0.009]. These group differences in the open-arm entries did not coincide with group differences in the number of closed-arm entries in the maze, which did not vary with MA history (Figure 4E; Dose X Sex ANOVA: all *p*-values > 0.079).

#### 2.2.4. Light–Dark Shuttle Box

A prior MA history also did not influence the latency to first enter the light side of the light–dark shuttle box (Figure 4F; Dose X Sex ANOVA: all *p*-values > 0.100), but it did alter the number of light-side entries by both male and female mice (Figure 4G) [Dose effect: F(1,116) = 3.529, *p* = 0.017; other *p*-values > 0.177]. This Dose effect reflected a difference between the MA-0.8 and MA-3.2 mice (Tukey’s test: *p* = 0.016), with no water–MA differences detected for this variable (Tukey’s tests: all *p*-values > 0.075). Similarly, a Dose effect was also detected for the time spent in the light side of the light–dark box (Figure 4H) [F(1,116) = 4.521, *p* = 0.005; Sex effect and interaction, *p*-values > 0.121], but for this variable, the Dose effect reflected a difference between the MA-1.6 and MA-3.2 mice (Tukey’s test: *p* = 0.004).

Taken together, these data from the elevated plus maze and light–dark shuttle-box tests fail to indicate a consistent effect of a prior subchronic history of MA upon agoraphobia/photophobia-like behavior during early withdrawal. 

#### 2.2.5. Forced-Swim Test

**Figure 5:**
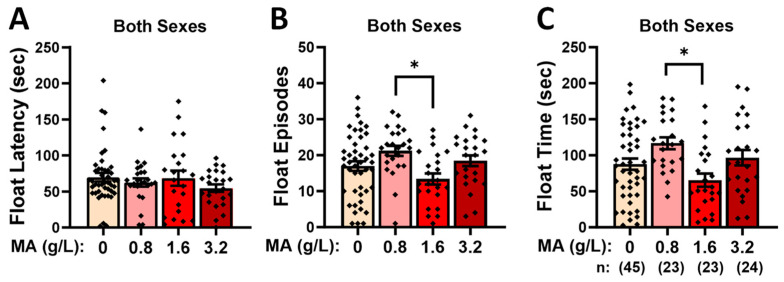
Summary of the effects of a 7-day history of oral MA (0, 0.8, 1.6, or 3.2 g/L) on (**A**) the latency to first float, (**B**), the number of floating episodes and (**C**) the time spent floating in the forced-swim test. The data represent the means ± SEMs of the numbers of mice indicated in parentheses in Panel (**C**). * *p* < 0.05 for specific dose comparison indicated (Tukey’s tests).

Prior MA history did not alter the latency to the first float in the forced-swim test (Figure 5A; Dose X Sex ANOVA: all *p*-values > 0.092), but it did influence both the number of floating episodes (Figure 5B) [Dose effect: F(1,116) = 3.910, *p* = 0.011] and the time spent floating (Figure 5C) [Dose effect: F(1,116) = 4.288, *p* = 0.007]. However, neither MA effect reflected any water–MA differences (*p*-values > 0.106) but rather reflected less floating by the MA-1.6 versus MA-0.8 mice (Tukey’s tests: for float number, *p* = 0.006; for float time, *p* = 0.003). These data fail to indicate an effect of prior MA history on the coping strategy in response to a swim stressor.

#### 2.2.6. Acoustic Startle and Prepulse Inhibition

**Figure 6:**
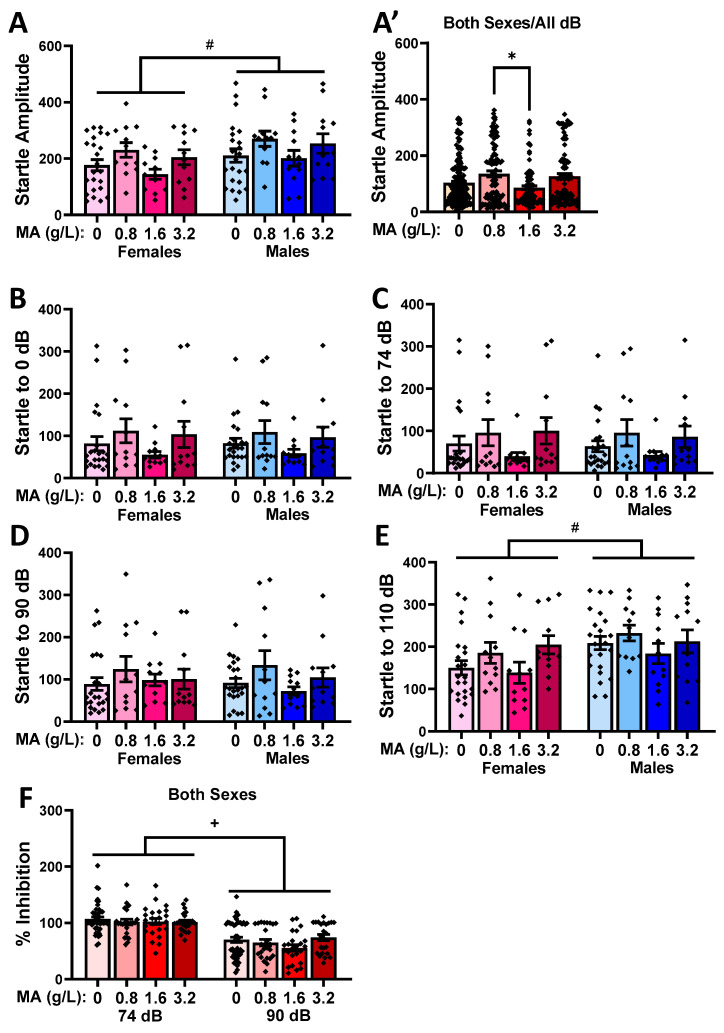
Summary of the effects of a 7-day history of oral MA (0, 0.8, 1.6, or 3.2 g/L) on acoustic startle (**A**–**E**) and prepulse inhibition (PPI) of acoustic startle (**F**). (**A**) Comparison of the average startle amplitude between male and female mice, with the different MA histories. (**A’**) Depiction of the main Dose effect for the average startle amplitude across the different acoustic stimuli. Depiction of the startle magnitude in response to the (**B**) 0 dB, (**C**) 74 dB, (**D**) 90 dB, and (**E**) 110 dB acoustic stimuli. (**F**) Depiction of the main Prepulse effect on PPI of acoustic startle. The data represent the means ± SEMs of the numbers of mice indicated in Figure 4. * *p* < 0.05 for specific dose comparison indicated (Tukey’s tests); # *p* < 0.05 for main effect of sex; + *p* < 0.05 for main effect of prepulse.

Overall, males exhibited a larger average startle amplitude than females in response to the repeated presentation of the 110 dB startle stimulus at the start of the session (Figure 6A, left vs. right) [Sex effect: F(1,117) = 5.716, *p* = 0.019]. While an overall Dose effect was detected for the startle amplitude during this habituation phase of the testing [F(1,117) = 3.209, *p* = 0.026; interaction: *p* = 0.970], in neither sex did a prior history of MA impact the startle amplitude, as indicated by no water–MA differences (Figure 6A′; Tukey’s tests: *p*-values > 0.104). The only group difference during the habituation phase was a difference between the MA-0.8 and MA-1.6 mice (Tukey’s test: *p* = 0.036; other *p*-values > 0.200). A sex difference was also noted for the magnitude of the startle elicited by the various acoustic stimuli (0, 74, 96, and 110 dB; Figure 6B–E) [Sex X Tone: F(3,330) = 4.812, *p* = 0.003], which reflected the higher startle magnitude in the males upon the 110 dB stimulus only (Figure 6E) [*t*-tests, for 110 dB: t(116) = 2.906, *p* = 0.004; for other stimuli: *p*-values > 0.746]. We also detected an overall effect of MA on the startle magnitude [Dose effect: F(1,110) = 2.899, *p* = 0.038; other *p*-values > 0.530], which again reflected a difference in the startle amplitude between the MA-0.8 and the MA-1.6 mice (Figure 6F; Tukey’s test: *p* = 0.046). In contrast to the startle response to single acoustic stimuli, we detected no sex or MA effects on the capacity of a 74 or 90 dB prepulse to inhibit the startle response to the 110 dB stimulus (Figure 6F) [Prepulse effect: F(1,110) = 155.47, *p* < 0.0001; other *p*-values > 0.151]. Taken together, these data for the acoustic startle and its prepulse inhibition indicate that a prior subchronic history of MA does not impact acoustic startle reactivity or sensorimotor gating.

### 2.3. Morris Water Maze

#### 2.3.1. Flag Test

**Figure 7:**
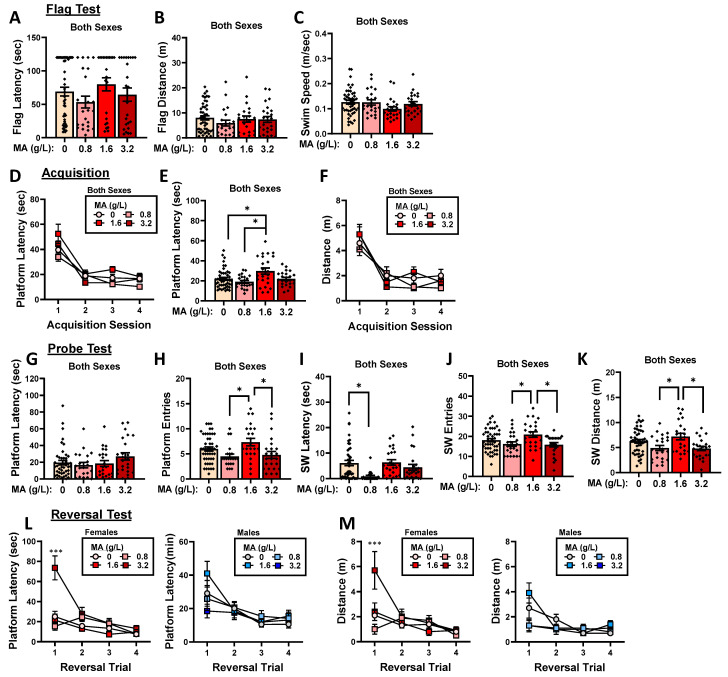
Summary of the effects of a 7-day history of oral MA (0, 0.8, 1.6, or 3.2 g/L) on spatial navigation in the Morris water maze assay, including visually cued navigation during the flag test (**A**–**C**), spatial learning during the acquisition phase of the assay (**D**–**F**), spatial recall during the probe test (**G**–**K**), and the reversal learning of a new platform location (**L**,**M**). These data represent the means ± SEMs of the numbers of mice indicated in parentheses in Figure 4. * *p* < 0.05 for specific dose comparison indicated (Tukey’s tests); *** *p* < 0.05 vs. three other MA doses (Tukey’s tests).

No group differences were noted for the time to locate a flagged platform (Figure 7A; Dose X Sex ANOVA: all *p*-values > 0.255), the distance traveled prior to locating the flagged platform (Figure 7B; Dose X Sex ANOVA: all *p*-values > 0.600), or the swim speed during the flag test (Figure 7C; Dose X Sex ANOVA: all *p*-values > 0.166). These data for the flag test argue that a prior subchronic history of oral MA does not alter visually cued spatial navigation or swimming ability.

#### 2.3.2. Acquisition

A prior history of MA self-administration did not alter the rate of the Morris water maze acquisition in either the female or male mice, as indexed by the latency to locate the hidden platform (Figure 7D) [Day effect: F(3,324) = 75.729, *p* < 0.0001; no interactions with the Day factor, *p*-values > 0.184]. However, an overall Dose effect was observed [F(3,108) = 4.398, *p* = 0.006], which reflected the longer latency of the MA-1.6 mice to locate the hidden platform relative to both the water controls and MA-0.8 mice (Figure 7E; Tukey’s tests, respectively: *p* = 0.025 and *p* = 0.006; other *p*-values > 0.740). Similar statistical results were obtained when the distance traveled prior to locating the hidden platform was considered as the dependent variable, although we did not detect any overall effects of a prior MA history for this measure (Figure 7F) [Day effect: F(3,324) = 53.343, *p* < 0.001; other *p*-values > 0.233]. These data do not support any systematic effect of prior subchronic oral MA history on spatial learning in the Morris water maze.

#### 2.3.3. Probe Test

We detected no group differences in the latency to first enter the former location of the hidden platform during the probe test (Figure 7G; Dose X Sex ANOVA: all *p*-values > 0.142), although we detected a significant Dose effect for the number of entries into the former platform location (Figure 7H) [Dose effect: F(3,116) = 4.739, *p* = 0.004; other *p*-values > 0.150]. As illustrated in Figure 7H, the effect of prior MA history on the number of platform zone entries did not vary systemically with the MA concentration; although it appeared that the MA-0.8 mice made fewer platform zone entries than the water controls, and no water–MA differences were detected (Tukey’s tests: *p*-values > 0.188), while the MA-1.6 mice entered the former platform zone more frequently than both the MA-0.8 and MA-3.2 mice (Tukey’s tests, respectively: *p* = 0.015 and *p* = 0.20). As alternate indices of recall, we also examined variables associated with the SW quadrant that formerly contained the platform. The MA-0.8 mice exhibited a shorter latency to enter the SW quadrant relative to the water controls (Figure 7I) [Dose effect: F(1,112) = 4.898, *p* = 0.003; other *p*-values > 0.642; Tukey’s test: *p* = 0.003], with no other group differences detected for this variable (Tukey’s tests: other *p*-values > 0.150). Prior MA history did not influence the total time spent in the SW quadrant (Dose X Sex ANOVA: all *p*-values > 0.312); however, Dose effects were observed for both the number of entries into this quadrant (Figure 7J) [Dose effect: F(3,115) = 3.768, *p* = 0.013; other *p*-values > 0.625] and the distance traveled in the SW quadrant (Figure 7K) [Dose effect: F(3,116) = 3.775, *p* = 0.013; other *p*-values > 0.768]. However, neither of these MA effects reflected differences between the MA-experienced mice and water controls (Tukey’s tests: for SW entries, *p*-values > 0.232; for SW distance, *p*-values > 0.202). Instead, the MA-1.6 mice entered the SW quadrant more times than both the MA-0.8 and MA-3.2 mice (Figure 7J; Tukey’s tests, respectively: *p* = 0.033 and *p* = 0.019), and they traveled a greater distance than both of these groups (Figure 7K; Tukey’s tests, respectively: *p* = 0.022 and *p* = 0.050). These data for the probe test fail to indicate any systematic effect of a prior subchronic MA history on spatial recall in the Morris water maze. 

#### 2.3.4. Reversal Learning

A significant three-way interaction was detected for the time course of reversal learning (Figure 7L) [F(9,327) = 4.787, *p* < 0.0001]. Deconstruction of the interaction along the Sex factor revealed a significant Trial X Dose interaction for females (Figure 7L, left) [F(8,168) = 6.562, *p* < 0.001], with no Dose effect or interaction detected in males (Figure 7L, right) [Trial effect: F(3,159) = 9.540, *p* < 0.001; other *p*-values > 0.471]. To decipher the source of the Dose X Trial interaction in females, the data were re-analyzed separately for each trial. A Dose effect was observed on Trial 1 [F(3,59) = 11.965, *p* < 0.0001], which reflected a longer latency to locate the repositioned platform by the MA-3.2 females versus all the other groups (Tukey’s tests: all *p*-values < 0.001). While the MA-3.2 females also exhibited the longest latency to locate the repositioned platform in Trial 2 of reversal learning [F(3,59) = 3.418, *p* = 0.023], their latency was not significantly different than those of the other groups (Tukey tests: all *p*-values > 0.072). No MA effect was detected for the latency of females to locate the repositioned platform in Trials 3 (Dose effect, *p* = 0.207) or 4 (Dose effect, *p* = 0.151). A similar pattern of results was obtained when the distance traveled prior to reaching the repositioned platform was considered (Figure 7M) [Dose X Sex X Trial: F(9,327) = 4.787, *p* < 0.0001; for females, Dose X Trial: F(9,168) = 6.562, *p* < 0.0001; for males, Trial effect: F(3,159) = 9.540, *p* < 0.0001; other *p*-values > 0.456]. Post hoc analyses conducted on the data for females indicated a greater distance traveled in Trial 1 by the MA-3.2 females versus all the other females (Figure 7M, left) [F(1,59) = 8.085, *p* < 0.0001; Tukey’s tests: *p*-values < 0.013; other *p*-values > 0.508], with no MA effects detected for Trials 2–4 (Dose X Trial ANOVAs: all *p*-values > 0.290). These data for reversal learning suggest that a subchronic history of higher MA concentrations can slow reversal learning, and it does so selectively in female mice. 

### 2.4. Radial-Arm Maze

**Figure 8:**
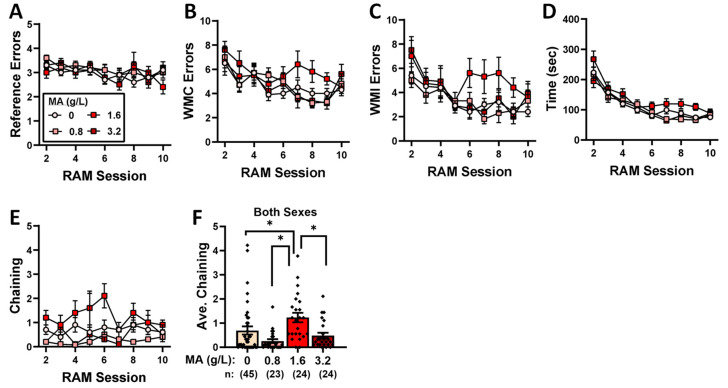
Summary of the effects of a 7-day history of oral MA (0, 0.8, 1.6, or 3.2 g/L) on behavior expressed during radial-arm maze (RAM) training. The data represent the means ± SEMs of the numbers of mice indicated in parentheses in Panel (**F**). * *p* < 0.05 for specific dose comparison indicated (Tukey’s tests).

We detected no group differences in the reduction in reference-memory errors exhibited by the mice on Days 2–9 of training (Figure 8A) [Session effect: F(8,808) = 2.539, *p* = 0.010; all other *p*-values > 0.455] or the number of working-memory correct errors (Figure 8B) [Session effect: F(8,808) = 7.047, *p* < 0.001; all other *p*-values > 0.12], the number of working-memory incorrect errors (Figure 8C) [Session effect: F(8,808) = 5.583, *p* < 0.0001; all other *p*-values > 0.135], or the total time taken to complete the radial-arm maze in each trial (Figure 8D) [Session effect: F(8,808) = 47.583, *p* < 0.0001; other *p*-values > 0.095]. Interestingly, we did detect a significant Dose effect for the number of chaining episodes (Figure 8E) [F(4,101) = 14.664, *p* < 0.0001; other *p*-values > 0.501], which reflected more chaining by the mice with a prior history of 1.6 g/L MA than all of the other groups tested (Tukey’s tests: 0 vs. 0.8, 1.6, and 3.2, *p*-values < 0.001; other *p*-values > 0.615). While these latter data suggest that the MA-1.6 mice employed a non-spatial strategy to navigate the radial-arm maze, the results from the radial-arm maze fail to indicate an effect of prior subchronic MA history upon working or reference memory.

### 2.5. Immunoblotting

A day following the 10th radial-arm maze session, the mice were rapidly decapitated, their brains were extracted, and the tissue from the ventral PFC, dorsal PFC, amygdala, and hippocampus was dissected (see Figure 1). Immunoblotting was then conducted to examine for changes in the expression of glutamate receptor-related proteins and some of their downstream effectors implicated in the neurobiology of substance abuse. 

#### 2.5.1. Glutamate Receptor-Related Proteins in the vPFC

**Figure 9:**
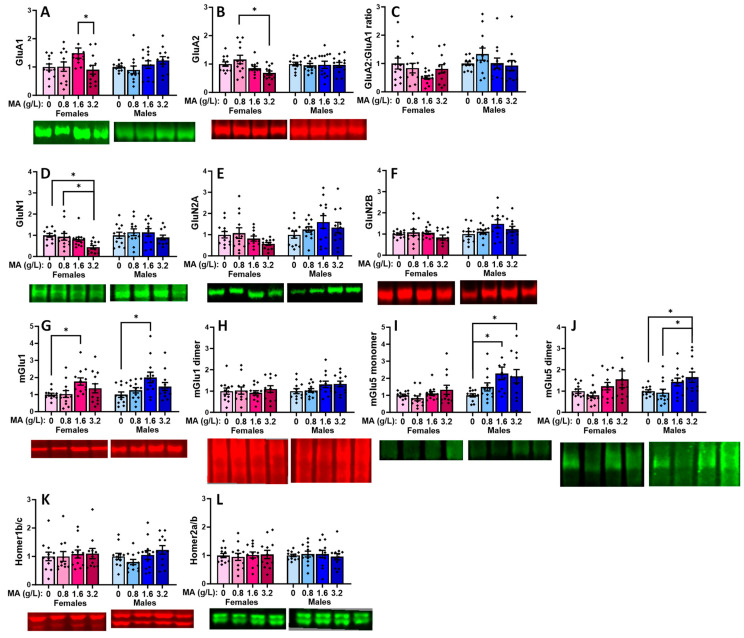
Summary of the effects of a 7-day history of oral MA (0, 0.8, 1.6, or 3.2 g/L) on the expressions of the following glutamate receptor-related proteins within the vPFC: the AMPA receptor subunits (**A**) GluA1, (**B**) GluA2, (**C**) the relative expression of GluA2 to GluA1, the NMDA receptor subunits (**D**) GluN1, (**E**) GluN2a, (**F**) GluN2b, the Group 1 mGlu receptors, (**G**) mGlu1 (monomer), (**H**) mGlu1 (dimer); (**I**) mGlu5 monomer, (**J**) mGlu5 dimer and the receptor scaffolding proteins, (**K**) Homer1b/c and (**L**) Homer2a/b. The data represent the means ± SEMs of 11–12 mice. * *p* < 0.05 for specific dose comparison indicated (Tukey’s tests).

In the vPFC, a prior history of oral MA did not alter the expression of either the GluA1 (Figure 9A) or GluA2 subunits of the AMPA receptor (Figure 9B), nor did it alter the relative expression of these receptor subunits in male mice (Figure 9C) [for GluA1: F(3,47) = 1.464, *p* = 0.237; for GluA2: F(1,47) = 0.45, *p* = 0.987; for ratio: F(3,47) = 1.114, *p* = 0.353]. In females, a history of 1.6 g/L MA elevated the GluA1 expression, relative to 3.2 g/L MA (Figure 9A) [F(3,47) = 2.901, *p* = 0.045; Tukey’s tests: MA-1.6 vs. MA-3.2, *p* = 0.49; other *p*-values > 0.127], while a history of 3.2 g/L MA reduced the GluA2 expression, relative to 0.8 g/L MA (Figure 9B) [F(1,47) = 4.324, *p* = 0.009; Tukey’s tests: MA-3.2 vs. MA-0.8, *p* = 0.007; other *p*-values > 0.115]. Although it appeared that the MA-1.6 females exhibited a lower relative expression of GluA2, this difference was not statistically significant owing to the variability in the data (Figure 9C) [F(1,45) = 1.944, *p* = 0.137]. A history of oral MA self-administration also lowered the expression of the GluN1 NMDA receptor subunit selectively in female mice (Figure 9D) [for females: F(1,45) = 4.476, *p* = 0.008; for males: F(1,45) = 0.604, *p* = 0.616], and this effect reflected the lower protein expression in the MA-3.2 females versus both the MA-0 and MA-0.8 females (Tukey’s tests: *p*-values < 0.024; other *p*-values > 0.126). Although similar female-selective, dose-dependent trends were observed for the GluN2A and GluN2B subunits of the NMDA receptor (Figure 9E,F), these dose effects were not statistically significant for either the female or male subjects [for GluN2A: females: F(1,47) = 2.049, *p* = 0.121; males: F(1,47) = 1.246, *p* = 0.305; for GluN2B: females: F(1,47) = 1.092, *p* = 0.362; males: F(1,47) = 2.281, *p* = 0.092].

Both the female and male MA-1.6 mice exhibited elevated mGlu1 monomer expression within the vmPFC, relative to the water-drinking controls (Figure 9G) [for females, F(3,47) = 3.146, *p* = 0.034; for males, F(1,47) = 3.649, *p* = 0.020; Tukey’s post hoc tests: for MA-0 vs. MA-1.6 females, *p* = 0.047, other *p*-values > 0.062; for MA-0 vs. MA-1.6 males, *p* = 0.014, other *p*-values > 0.098]. However, we failed to detect a change in the vPFC expression of the active mGlu1 dimer in females or males (Figure 9H) [for females, F(3,47) = 0.270, *p* = 0.847; for males, F(3,44) = 2.113, *p* = 0.112]. No MA effect was detected for the mGlu5 monomer (Figure 9I) or dimer (Figure 9J) expression in females (respectively, univariate ANOVA: *p* = 0.203 and *p* = 0.128). However, both the 1.6 and 3.2 g/L doses elevated the levels of the mGlu5 monomer in males (Figure 9I) [F(1,47) = 3.995, *p* = 0.013; Tukey’s: MA-0 vs. MA-1.6 and MA-3.2, *p* = 0.018 and *p* = 0.050, respectively], while the 3.2 g/L dose elevated the mGlu5 dimer expression relative to both the 0 and 0.8 g/L doses in males (Figure 9J) [F(3,47) = 3.980, *p* = 0.014; Tukey’s: MA-3.2 vs. MA-0 and MA-0.8, *p* = 0.050 and *p* = 0.026, respectively]. The effects of the MA self-administration on the glutamate receptor expression did not overtly relate to changes in the expression of its major scaffolding proteins Homer1b/c (Figure 9K; univariate ANOVAs: for females, *p* = 0.954; for males, *p* = 0.147] or Homer2a/b (Figure 9L; univariate ANOVAs: for females, *p* = 0.960; for males, *p* = 0.902). 

#### 2.5.2. Downstream Effectors in the vPFC

**Figure 10:**
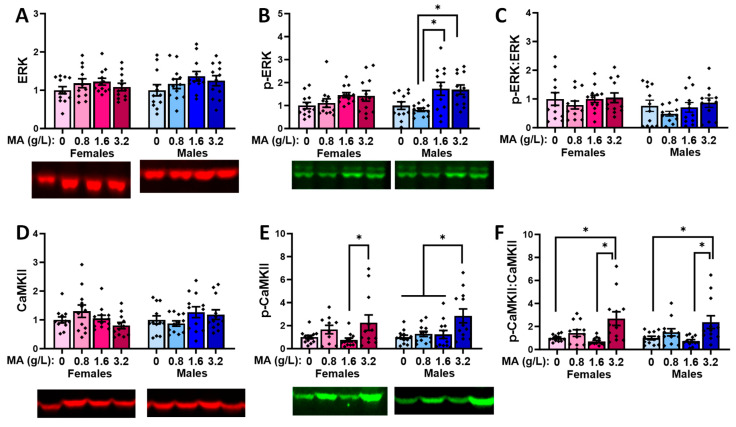
Summary of the effects of a 7-day history of oral MA (0, 0.8, 1.6, or 3.2 g/L) on the expression and phosphorylation state of ERK (**A**,**B**) and CaMKII (**D**,**E**) within the vPFC. The relative expression of p-ERK to ERK (**C**) and p-CaMKII to CaMKII (**F**) are also depicted. The data represent the means ± SEMs of 11–12 mice. * *p* < 0.05 for specific dose comparison indicated (Tukey’s tests).

Oral MA history did not alter the vmPFC levels of ERK in either sex (Figure 10A; univariate ANOVAs: for females, *p* = 0.398; for males, *p* = 0.264). The two highest MA concentrations elevated the p-ERK expression in the males, relative to the MA-0.80 males (Figure 10B) [F(1,47) = 5.820, *p* = 0.002; Tukey’s tests: *p*-values < 0.015; other *p*-values > 0.053], with no MA effect detected in the females (Figure 10B; F(1,47) = 1.759, *p* = 0.169]. However, when the ratio of the phosphorylated to total ERK expression was considered, we detected no MA effects in either sex (Figure 10C; univariate ANOVAs: for females, *p* = 0.919; for males, *p* = 0.425), indicating that a brief history of oral MA intake does not alter the activational state of ERK in the vmPFC. Neither females nor males exhibited changes in the total expression of CaMKII (Figure 10D; univariate ANOVAs: for females, *p* = 0.124; for males, *p* = 0.267). However, the highest MA dose elevated the p-CaMKII expression in the males, relative to all the other groups (Figure 10E) [F(1,47) = 5.086, *p* = 0.004; Tukey’s tests: *p*-values < 0.026; other *p*-values > 0.953]. The MA-3.2 females also exhibited higher p-CaMKII expression [F(1,45) = 2.908, *p* = 0.046], but this effect reflected a difference between the MA-3.2 and MA-1.6 mice only (Figure 10E; Tukey’s tests: *p* = 0.049; other *p*-values > 0.129). When the ratio of the phosphorylated to total CaMKII expression was considered (Figure 10F), a more robust dose–effect was apparent for females [F(1,47) = 6.643, *p* = 0.0001], which reflected a higher relative expression of p-CaMKII in the MA-3.2 females versus the MA-0 (*p* = 0.006) and MA-1.6 (*p* = 0.001; for MA-3.2 vs. MA-1.6, *p* = 0.057) females. In males, the MA effect [F(1.47) = 4.658, *p* = 0.007] reflected similar group differences (Figure 10F; Tukey’s: MA-3.2 vs. MA-0 mice, *p* = 0.027; MA-3.2 vs. MA-1.6 mice, *p* = 0.006; other *p*-values > 0.250). Thus, in contrast to ERK, a brief history of oral MA self-administration increases the activational state of CaMKII in the vmPFC.

#### 2.5.3. Glutamate Receptor-Related Proteins in the dPFC

**Figure 11:**
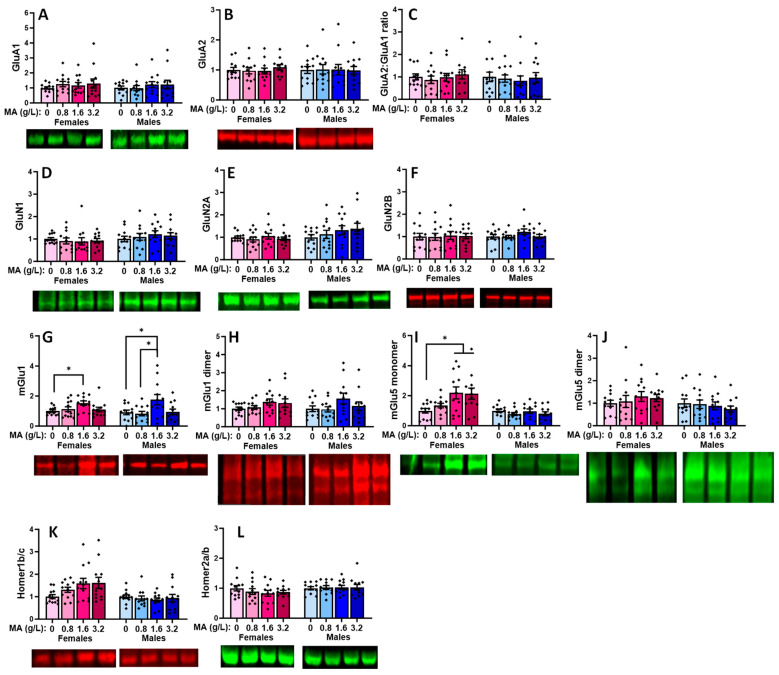
Summary of the effects of a 7-day history of oral MA (0, 0.8, 1.6, or 3.2 g/L) on the expressions of the following glutamate receptor-related proteins within the dPFC: the AMPA receptor subunits (**A**) GluA1, (**B**) GluA2, (**C**) the relative expression of GluA2 to GluA1, the NMDA receptor subunits (**D**) GluN1, (**E**) GluN2a, (**F**) GluN2b, the Group 1 mGlu receptors, (**G**) mGlu1 (monomer), (**H**) mGlu1 (dimer); (**I**) mGlu5 monomer, (**J**) mGlu5 dimer and the receptor scaffolding proteins, (**K**) Homer1b/c and (**L**) Homer2a/b. The data represent the means ± SEMs of 11–12 mice. * *p* < 0.05 for specific dose comparison indicated (Tukey’s tests).

In the dPFC, we failed to detect any MA-induced changes in the expression of GluA1 (Figure 11A; one-way ANOVAs: for females, *p* = 0.768; for males, *p* = 0.440), GluA2 (Figure 11B; one-way ANOVAs: for females, *p* = 0.799; for males, *p* = 0.998), or their relative expression (Figure 11C; one-way ANOVAs: for females, *p* = 0.716; for males, *p* = 0.780). Similarly, we did not detect any MA-induced changes in the expression of GluN1 (Figure 11D; one-way ANOVAs: for females, *p* = 0.928; for males, *p* = 0.752), GluN2A (Figure 11E; one-way ANOVAs: for females, *p* = 0.734; for males, *p* = 0.477), or GluN2B (Figure 11F; one-way ANOVAs: for females, *p* = 0.991; for males, *p* = 0.293). Thus, in contrast to the vPFC, we detected no effects of a prior history of oral MA upon the expression of any of the iGluR subunits examined within the dPFC.

That being said, we did detect a significant MA effect on the dPFC expression of the mGlu1 monomer in both female and male mice (Figure 11G) [for females, F(3,47) = 2.908, *p* = 0.045; for males, F(3,47) = 3.748, *p* = 0.018]. In females, this MA effect reflected higher mGlu1 monomer expression in the MA-1.6 versus MA-0 mice (*p* = 0.045), with no other group differences observed (*p*-values > 0.142), while, in males, the mGlu1 monomer expression was significantly higher in the MA-1.6 mice, relative to both the MA-0 (*p* = 0.050) and MA-0.8 (*p* = 0.026) mice (other *p*-values > 0.064). In contrast, we detected no effects on the dPFC expression of the active mGlu1 dimer in either sex (Figure 11H) [for females, F(3,47) = 1.278, *p* = 0.294; for males, F(3,47) = 1.628, *p* = 0.196]. Neither male nor female mice exhibited any changes in the dPFC expression of either the mGlu5 monomer (Figure 11I) or dimer (Figure 11J; one-way ANOVAs: for female monomer, *p* = 0.127; for female dimer, *p* = 0.747; for male monomer, *p* = 0.505; for male dimer, *p* = 0.756). Although an inspection of Figure 11K suggested a dose-dependent increase in the Homer1b/c expression within the dPFC of the female mice, this effect did not reach statistical significance [F(3,47) = 2.319, *p* = 0.089], nor did we detect any change in the Homer1b/c in the males (one-way ANOVA: *p* = 0.817). Likewise, we detected no MA effect on the dPFC expression of Homer2a/b (Figure 11L; one-way ANOVAs: for females, *p* = 0.550; for males, *p* = 0.735). 

#### 2.5.4. Downstream Effectors in the dPFC

**Figure 12:**
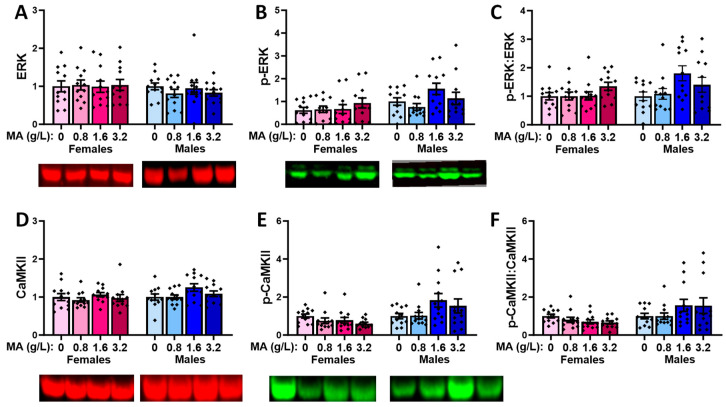
Summary of the lack of any significant effects of a 7-day history of oral MA (0, 0.8, 1.6, or 3.2 g/L) on the expression and phosphorylation state of ERK (**A**,**B**) and CaMKII (**D**,**E**) within the dPFC. The relative expression of p-ERK to ERK (**C**) and p-CaMKII to CaMKII (**F**) are also depicted. The data represent the means ± SEMs of 11–12 mice.

MA-experienced female mice exhibited no significant changes in the expression or activational state of ERK in the dPFC (one-way ANOVAs: F(3,47) < 1.528, *p*-values > 0.220], while a non-significant trend towards an increase in the relative expression of p-ERK was detected in the males (Figure 12A–C) [one-way ANOVAs: F(3,47) < 2.748, *p*-values > 0.053]. No MA effects were detected for the expression or activational state of CaMKII within the dPFC of either the female or male mice (Figure 12D–F; one-way ANOVAs: for females, *p*-values > 0.137; for males, *p*-values > 0.092).

#### 2.5.5. Glutamate Receptor-Related Proteins in the Hippocampus

**Figure 13:**
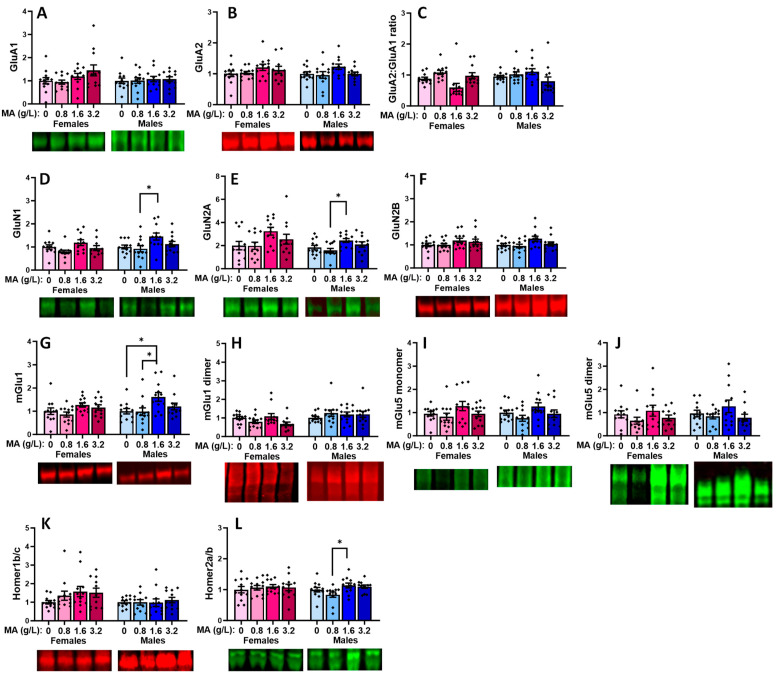
Summary of the effects of a 7-day history of oral MA (0, 0.8, 1.6, or 3.2 g/L) on the expressions of the following glutamate receptor-related proteins within the hippocampus: the AMPA receptor subunits (**A**) GluA1, (**B**) GluA2, (**C**) the relative expression of GluA2 to GluA1, the NMDA receptor subunits (**D**) GluN1, (**E**) GluN2a, (**F**) GluN2b, the Group 1 mGlu receptors, (**G**) mGlu1 (monomer), (**H**) mGlu1 (dimer); (**I**) mGlu5 monomer, (**J**) mGlu5 dimer and the receptor scaffolding proteins, (**K**) Homer1b/c and (**L**) Homer2a/b. The data represent the means ± SEMs of 11–12 mice. * *p* < 0.05 for specific dose comparison indicated (Tukey’s tests).

No MA effect was detected for the hippocampal expression of GluA1, GluA2, or their relative expression in either female [for GluA1: F(1.46) = 2.130; *p* = 0.110; for GluA2: [F(1,67) = 1.122, *p* = 0.351; for GluA2:GluA1 ratio: F(1,46) = 1.329, *p* = 0.277] or male (Figure 13A–C) [for GluA1: F(1,47) = 0.121, *p* = 0.948; for GluA2: F(1,47) = 2.220, *p* = 0.099; for GluA2:GluA1 ratio: F(1,47) = 1.276, *p* = 0.294] mice. Females exhibited a non-significant MA effect for hippocampal GluN1 expression [F(1,47) = 2.694, *p* = 0.058], which reflected a difference between the MA-0.8 vs. -1.6 mice (Figure 13D; *p* = 0.035). In males, this specific MA effect was statistically significant [F(1,47) = 3.496, *p* = 0.023; MA-0.8 vs. -1.6: *p* = 0.023), while the apparent difference between the MA-1.6 and MA-0 males was not (Figure 13D; Tukey’s test: *p* = 0.063). In contrast, no MA effect was detected on the GluN2B expression for either female or male mice (Figure 13F) [for females, F(1,47) = 1.192, *p* = 0.324; for males, [F(1,47) = 2.577, *p* = 0.066]. A similar sex-selective pattern of MA effects was detected for the hippocampal expression of the GluN2A subunit, with females exhibiting a non-significant trend for an MA effect (Figure 13E) [F(1,47) = 2.729, *p* = 0.055; Tukey’s Tests: 0 vs. 1.6 (*p* = 0.08); 0.8 vs. 1.6 (*p* = 0.077)], and a significant difference was detected between the MA-0.8 and MA-1.6 males [F(1,47) = 3.624, *p* = 0.020; Tukey’s test: *p* = 0.016], but there was no difference between the MA-0 and other MA doses (Tukey’s tests: *p* > 0.125).

A male-selective effect of MA was also detected for hippocampal mGlu1 expression that reflected a difference between the MA-1.6 mice and both the MA-0 and MA-0.8 mice (Figure 13G) [for females, F(1,47) = 2.461, *p* = 0.075; for males, F(1,47) = 3.646, *p* = 0.020; Tukey’s tests for males: 0 vs. 1.6, *p* = 0.034; 0.8 vs. 1.6, *p* = 0.029; other *p*-values > 0.098]. In the case of the mGlu1 dimer, females exhibited a statistically non-significant MA effect on the protein expression (Figure 13H) [F(3,47) = 2.796, *p* = 0.051], while males exhibited no evidence of any MA effect [F(3,47) = 0.639, *p* = 0.594]. Although it appeared that a history of MA-1.6 elevated the hippocampal expression of both the monomer and dimer forms of mGlu5 (Figure 13I–J), no significant MA effects were detected for either protein in the female [for monomer, F(1,47) = 1.818, *p* = 0.158; for dimer, F(1,45) = 1.359, *p* = 0.268] or male [for monomer, F(1,47) = 2.069, *p* = 0.118; for dimer, F(1,47) = 1.818, *p* = 0.158] mice, likely owing to the large variability in the data. No significant MA effect was detected for Homer1b/c in either the female or male mice (Figure 13K) [for females, F(3,46) = 1.324, *p* = 0.279; for males, F(3,46) = 0.191, *p* = 0.902], and females also failed to exhibit an MA effect on the hippocampal Homer2a/b expression [F(1,47) = 0.231, *p* = 0.874]. In contrast, a difference was observed between the MA-0.8 and MA-1.6 males with respect to the Homer2a/b expression (Figure 13L) [F(1,47) = 3.290, *p* = 0.029; Tukey’s tests: 0.8 vs. 1.6, *p* = 0.029].

#### 2.5.6. Downstream Effectors in the Hippocampus

**Figure 14:**
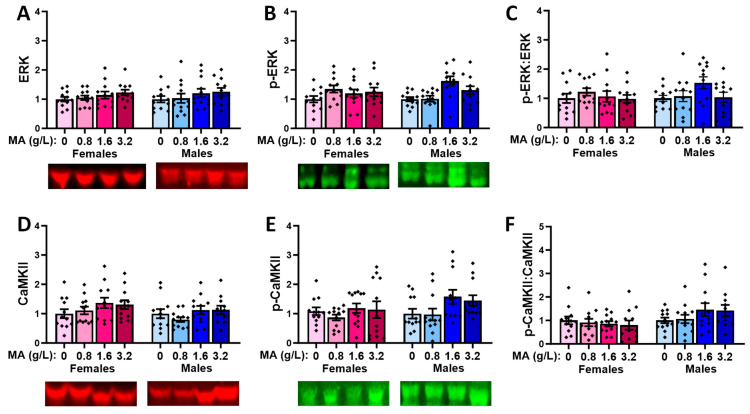
Summary of the effects of a 7-day history of oral MA (0, 0.8, 1.6, or 3.2 g/L) on the expression and phosphorylation state of ERK (**A**,**B**) and CaMKII (**D**,**E**) within the hippocampus. The relative expression of p-ERK to ERK (**C**) and p-CaMKII to CaMKII (**F**) are also depicted. The data represent the means ± SEMs of 11–12 mice.

A prior brief history of oral MA did not alter the total ERK protein expression in the hippocampus of either female or male mice (Figure 14A) [for females, F(1,47) = 1.103, *p* = 0.358; for males, F(1,47) = 1.752, *p* = 0.170]. Although females did not exhibit an MA-induced change in the hippocampal p-ERK levels (Figure 14B) [F(1.47) = 1.270, *p* = 0.297], a robust MA effect was detected in the males that reflected the higher p-ERK expression in the MA-1.6 mice versus both the MA-0 and MA-0.8 mice (Figure 14B) [F(1,47) = 5.601, *p* = 0.002; Tukey’s tests: 0 vs. 1.6, *p* = 0.005; 0.8 vs. 1.6, *p* = 0.006]. However, we detected no significant effect of MA on the relative hippocampal expression of p-ERK (Figure 14C) [for females, F(1,47) = 0.499, *p* = 0.685; for males, F(1,47) = 2.156, *p* = 0.107]. 

No MA-related changes were detected regarding the hippocampal expression of CaMKII (Figure 14D) [for females, F(1,47) = 1.232, *p* = 0.309; for males, F(1,47) = 1.387, *p* = 0.260], p-CaMKII (Figure 14E) [for females, F(1.47) = 0.299, *p* = 0.826; for males, F(1,47) = 2.457, *p* = 0.075], or their relative expression (Figure 14F) [for females, F(1,47) = 0.277, *p* = 0.842; for males, F(1,47) = 1.240, *p* = 0.307]. 

#### 2.5.7. Glutamate-Related Proteins within the Amygdala

**Figure 15:**
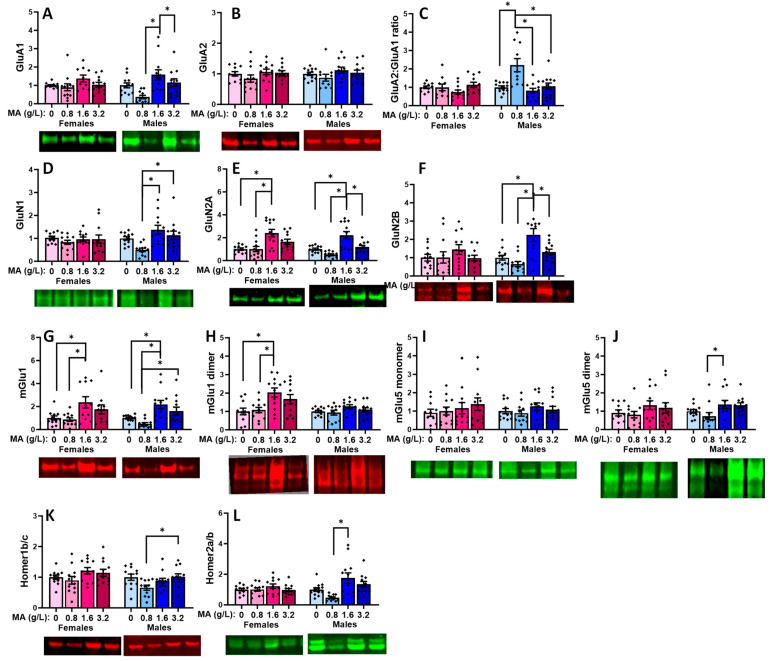
Summary of the effects of a 7-day history of oral MA (0, 0.8, 1.6, or 3.2 g/L) on the expressions of the following glutamate receptor-related proteins within the amygdala: the AMPA receptor subunits (**A**) GluA1, (**B**) GluA2, (**C**) the relative expression of GluA2 to GluA1, the NMDA receptor subunits (**D**) GluN1, (**E**) GluN2a, (**F**) GluN2b, the Group 1 mGlu receptors, (**G**) mGlu1 (monomer), (**H**) mGlu1 (dimer); (**I**) mGlu5 monomer, (**J**) mGlu5 dimer and the receptor scaffolding proteins, (**K**) Homer1b/c and (**L**) Homer2a/b. The data represent the means ± SEMs of 11–12 mice. * *p* < 0.05 for specific dose comparison indicated (Tukey’s tests).

In contrast to the other brain regions, we detected a significant MA effect for the amygdala expression of GluA1 in the male subjects but not in the female subjects (Figure 15A) [for females, F(3,44) = 1.852, *p* = 0.153; for males, F(3,46) = 6.689, *p* < 0.001]. Tukey’s tests revealed that the GluA1 expression was lower in the MA-0.8 males, relative to both the MA-1.6 (*p* = < 0.001) and MA-3.2 (*p* = 0.030) mice. No MA effect on the GluA2 expression was apparent in either (Figure 15B; for females, F(3,46) = 1.189, *p* = 0.325; for males, F(3,46) = 1.227, *p* = 0.312). However, an examination of the relative expression of these AMPA receptor subunits indicated a male-selective effect (Figure 15C) [for females, F(3,44) = 1.897, *p* = 0.145; for males, F(3,45) = 9.350, *p* < 0.001]. In males, the MA effect reflected a higher GluA2:GluA1 ratio for the MA-0.8 versus MA-0 (*p* < 0.001), MA-1.6 (*p* < 0.001), and MA-3.2 (*p* = 0.001) mice. MA-induced changes in the amygdala GluN1 expression were also male-selective (Figure 15D) [for females, F(3.46) = 0.47, *p* = 0.700; for males, F(3,46) = 6.437, *p* = 0.001] and reflected the lower GluN1 expression in the MA-0.8 males relative to the MA-1.6 (*p* < 0.001) and MA-3.2 (*p* = 0.018) males. In contrast to the male-selective effect of the MA on the AMPA receptor subunit expression in the amygdala, we detected changes in GluN2A in both the female [F(3,46) = 7.840, *p* < 0.001] and male [F(3,44)-13.436, *p* < 0.001] mice. As illustrated in Figure 15E, the MA effect in females reflected the higher GluN2A expression in the MA-1.6 versus MA-0 (*p* < 0.001) and MA-0.8 (*p* < 0.001) mice, while, in males, the MA effect reflected the higher GluN2A expression in the MA-1.6 mice relative to the other three MA doses (MA-1.6 vs. MA-0, *p* < 0.001, MA-0.8, *p* < 0.001, and MA-3.2, *p* = 0.005). Females failed to exhibit any MA effect on the GluN2B expression within the amygdala (Figure 15F) [F(3,45) = 0.599, *p* = 0.620), while males exhibited a robust MA effect [F(3,46) = 9.867, *p* < 0.001] that reflected higher GluN2B levels in the MA-1.6 males relative to the other three MA doses (Tukey’s tests: MA-1.6 vs. MA-0, *p* = 0.002, MA-0.8, *p* < 0.001, and MA-3.2, *p* = 0.015). 

Both sexes exhibited MA-induced changes in the amygdala expression of the mGlu1 monomer (Figure 15G) [for females, F(3,44) = 4.608, *p* = 0.007; for males, F(3,44) = 7.821, *p* < 0.001]. For females, the MA effect reflected the higher mGlu1 expression in the MA-1.6 versus both the MA-0 (*p* = 0.027) and MA-0.8 (*p* = 0.011) mice, while, in males, the MA effect reflected the higher expression in the MA-1.6 mice, relative to both the MA-0 (*p* = 0.011) and MA-0.8 (*p* < 0.001) mice, in addition to the higher mGlu1 expression in the MA-3.2 versus MA-0.8 mice (*p* = 0.022). However, only females exhibited MA-induced changes in the expression of the active mGlu1 dimer (Figure 15H) [for females, F(3,46) = 4.992, *p* = 0.005; for males, F(3,46) = 2.070, *p* = 0.118], and this female-selective MA effect reflected the higher mGlu1 dimer expression in the MA-1.6 versus MA-0 (*p* = 0.013) and MA-0.8 (*p* = 0.021) mice. Although the mGlu1 dimer levels were also relatively high in the MA-3.2 mice, the group differences were not statistically significant (Tukey’s tests: *p*-values > 0.115). In contrast to mGlu1, neither females nor males exhibited any change in the amygdala expression of the monomer form of mGlu5 (Figure 15I) [for females, F(3,46) = 0.570, *p* = 0.638; for males, F(3,46) = 0.751, *p* = 0.528]. Females also failed to exhibit any MA-related change in the expression of the mGlu5 dimer (Figure 15J) [F(3,46) = 1.152, *p* = 0.339], while an MA effect was detected in males [F(3,47) = 3.160, *p* = 0.034], which reflected a modest difference between the MA-0.8 and MA-1.6 mice (*p* = 0.05). In female mice, the MA-related changes in the amygdala mGlu1 expression were not accompanied by changes in Homer1b/c (Figure 15K) [F(3,46) = 1.739, *p* = 0.173] or Homer2a/b (Figure 15L) [F(3,46) = 1.061, *p* = 0.376]. In contrast, males exhibited a difference in Homer1b/c (Figure 15K) [F(3,46) = 3.103, *p* = 0.036], which reflected the higher protein expression in the MA-3.2 versus M-0.8 mice only (*p* = 0.045), in addition to a difference in Homer2a/b (Figure 15L) [F(3,46) = 5.099, *p* = 0.004], which reflected the higher protein expression in the MA-1.6 versus MA-0.8 mice only (*p* = 0.003). 

#### 2.5.8. Downstream Effectors in Amygdala

**Figure 16:**
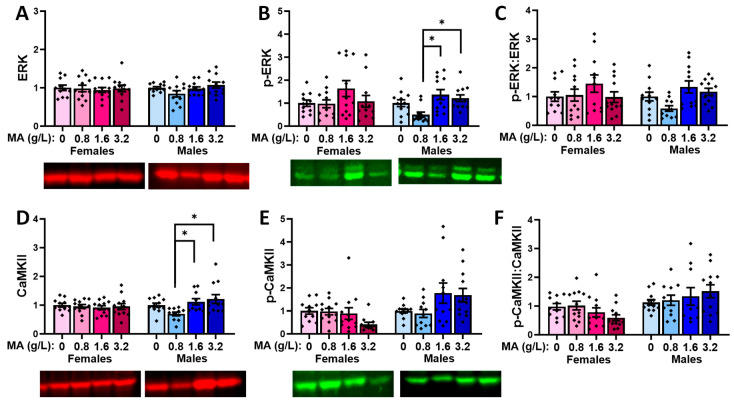
Summary of the effects of a 7-day history of oral MA (0, 0.8, 1.6, or 3.2 g/L) on the expression and phosphorylation state of ERK (**A**,**B**) and CaMKII (**D**,**E**) within the amygdala. The relative expression of p-ERK to ERK (**C**) and p-CaMKII to CaMKII (**F**) are also depicted. The data represent the means ± SEMs of 11–12 mice. * *p* < 0.05 for specific dose comparison indicated (Tukey’s tests).

No MA effects were detected for the total or relative protein expression of ERK and p-ERK within the amygdala of the female mice (Figure 16A–C) [for ERK, F(3,46) = 0.101, *p* = 0.959; for p-ERK, F(3,45) = 1.628, *p* = 0.197; for ratio, F(3,45) = 1.736, *p* = 0.174]. Although males did not exhibit an MA effect on the total ERK expression within the amygdala (Figure 16A) [F(3,46) = 2.193, *p* = 0.103], an MA effect was detected for both the total p-ERK (Figure 16B) [F(3,46) = 5.264, *p* = 0.004] and relative p-ERK (Figure 16C) [F(3,46) = 4.228, *p* = 0.010] expression. For p-ERK, the MA-0.8 males exhibited a lower expression than both the MA-1.6 (*p* = 0.003) and MA-3.2 (*p* = 0.018) males, while a similar group difference was detected for the relative p-ERK expression (MA-0.8 vs. MA-1.6, *p* = 0.008, vs. MA-3.2, *p* = 0.055). 

Female mice did not exhibit any MA effects on the total protein expression of CaMKII in the amygdala (Figure 16D) [F(3,46) = 0.209, *p* = 0.890], while males exhibited MA-induced differences in the CaMKII expression [F(3,44) = 6.518, *p* = 0.008] that reflected the lower expression in the MA-0.8 males versus both the MA-1.6 (*p* = 0.045) and MA-3.2 (*p* = 0.006) males. Only statistical trends towards a MA effect were observed for the amygdala expression of p-CaMKII in both females and males (Figure 16E) [for females, F(3,46) = 2.664, *p* = 0.060; for males, F(3,45) = 2.713, *p* = 0.057], and although the analysis of the relative expression of p-CaMKII indicated an MA effect in the female amygdala (Figure 16F) [F(3,46) = 2.946, *p* = 0.043], post hoc analyses did not indicate any significant group differences (Tukey’s tests: *p*-values > 0.052). No MA effect was detected for the relative p-CaMKII expression within the amygdala of the males (Figure 16F) [F(3,45) = 1.073, *p* = 0.371]. 

### 2.6. Behavioral Pharmacological Studies

#### 2.6.1. Effects of Systemic Inhibition of mGlu1 on Measures of Negative Affect

To probe the functional relevance of elevated mGlu1 expression (see Table 1) for the MA-induced increase in the measures of negative affect, we conducted a follow-up study in the male and female mice with a 1-week history of 3.2 g/L MA self-administration, during which we determined the effect of pretreatment (30 min earlier) with 5 mg/kg of the mGlu1-negative allosteric modulator JNJ 16259685 on the behavior expressed in the marble-burying, elevated plus maze, and light–dark shuttle-box tests. These tests were selected based on the observations from the original behavioral test battery that indicated MA effects (Figure 3 and Figure 4). 

**Figure 17:**
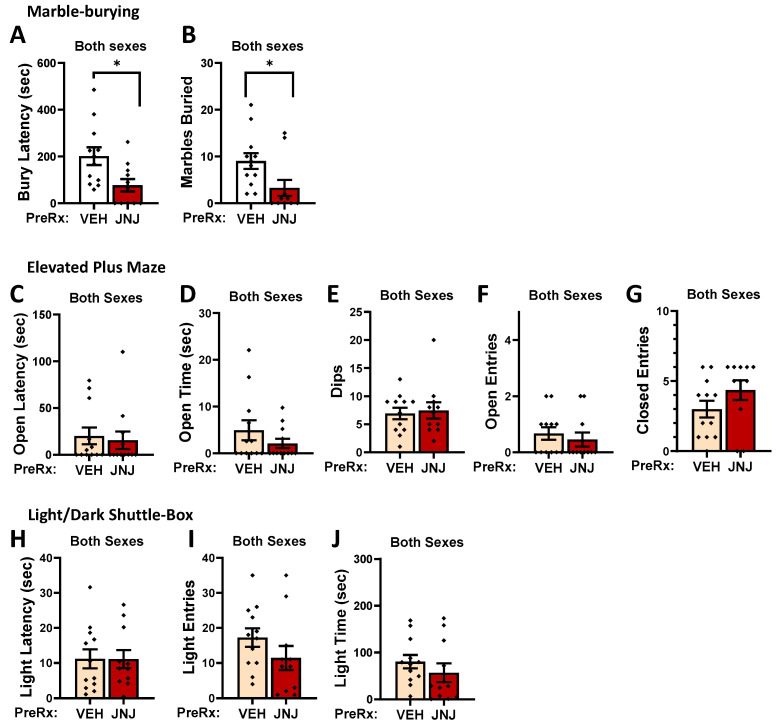
Summary of the effects of pretreatment with 5 mg/kg JNJ 16259685 (JNJ) or vehicle (VEH) on behavior expressed by MA-3.2 mice under marble-burying (**A**,**B**), elevated plus maze (**C**–**G**), and light–dark shuttle-box (**H**–**J**) procedures. The data represent the means ± SEMs of 11–12 mice (5–6 males/6 females). * *p* < 0.05 (*t*-tests).

A comparison of the average MA intake exhibited by the mice prior to anxiety testing indicated no group differences (Pretreatment X Sex ANOVA: all *p*-values > 0.538; for female VEH: 31.52 ± 3.01; for female JNJ: 38.78 ± 9.99; for male VEH: 34.30 ± 5.28; for male JNJ: 37.62 ± 12.95 mg/kg). Thus, we did not detect a sex difference in the MA intake in this follow-up study. Further, no sex differences were detected for any of the anxiety measures as determined by Pretreatment X Sex ANOVAs (*p*-values > 0.136). Thus, the data were collapsed across the Sex factor for visualization, resulting in final samples sizes of 12 for the VEH-pretreated animals and 11 for the JNJ-pretreated animals. The JNJ pretreatment reduced both the latency to begin burying marbles (Figure 17A) [t(19) = 2.569, *p* = 0.005] as well as the number of marbles buried by the MA-experienced mice (Figure 17B) [t(19) = 2.012, *p* = 0.031]. However, no JNJ effects were detected for any of the measures in the elevated plus maze (Figure 17C–G; *t*-tests: *p*-values > 0.262) or light–dark shuttle-box (Figure 17H–J; *t*-tests: *p*-values > 0.121). These data argue that MA-induced mGlu1 hyperactivity contributes to some aspects of negative affect observed during early MA withdrawal. 

#### 2.6.2. Effects of NMDA Receptor Manipulation on Reversal Learning in Morris Water Maze

**Figure 18:**
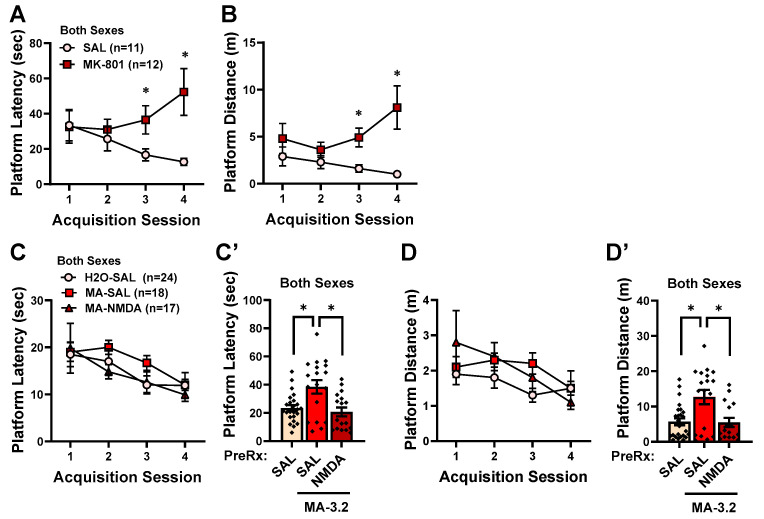
Summary of the effects of pretreatment with 0.1 mg/kg MK-801 (**A**,**B**) or 25 mg/kg NMDA (**C**,**D**) on reversal learning in the Morris water maze. The data represent the numbers of mice indicated in parentheses. * *p* < 0.05 (*t*-tests for Panels (**A**,**B**); Tukey’s tests for Panels (**C**′,**D**′)).

We first examined whether a reduction in the NMDA receptor function was sufficient to mimic the MA-induced deficit in the reversal learning exhibited by females in the Morris water maze by assaying the effects of systemic pretreatment with 0.1 mg/kg MK-801 on reversal learning in MA-naive mice. To ensure that the mice slated for pretreatment with saline versus MK-801 exhibited equivalent spatial memories following Morris maze acquisition, we compared their behavior in the probe test. Not shown, we detected no differences between the mice slated to receive saline (SAL) versus MK-801 (MK-801) on any measure during the probe test (Sex X Pretreatment ANOVAs: from latency to former location, *p*-values > 0.216; entries into the platform zone, *p*-values > 0.067; time in former quadrant, *p*-values > 0.429). However, when pretreated with MK-801, the MA-naïve mice exhibited a deficit in reversal learning (Figure 18A). This was supported by a significant Pretreatment X Trial interaction for the latency in finding the relocated platform [F(3,66) = 2.832, *p* = 0.045], which reflected an MK-801 effect on Trials 3 and 4 (Figure 18A) [respectively, t(24) = 2.299, *p* = 0.031 and t(24) = 2.942, *p* = 0.007; for Trials 1–2, *p*-values > 0.276]. The pattern of MK-801 effects were similar, although less statistically robust, for the distance traveled prior to locating the repositioned platform (Figure 18B) [Pretreatment X Trial: F(3,66) = 2.653, *p* = 0.056].

We next tested whether systemic pretreatment with the NMDA receptor agonist NMDA could reverse the reversal deficit observed in the MA-experienced mice. A water-drinking, MA-naïve control group (H2O-SAL) was included in this study to confirm that prior MA experience induced a reversal-learning impairment. The intake of 3.2 g/L MA in this study was comparable to that exhibited in the original study (see Figure 1), and we detected no difference in the MA intake between the mice slated to receive the saline vehicle (MA-SAL) or 25 mg/kg NMDA (MA-NMDA) [Pretreatment X Sex ANOVA: all *p*-values > 0.354; for female MA-SAL: 29.82 ± 3.38 (*n* = 9); *p* for female MA-NMDA: 38.61 ± 6.62 (*n* = 8); *p* for male MA-SAL: 29.07 ± 3.63 (*n* = 9); *p* for male MA-NMDA: 30.78 ± 7.68 (*n* = 9)]. Further, no differences were observed between the MA-SAL, MA-NMDA, and H2O-SAL mice with respect to performance during the probe test (Sex X Group ANOVAs: latency to former location, *p*-values > 0.0.104; entries into the platform zone, *p*-values > 0.776; time in former quadrant, *p*-values > 0.103). Thus, the MA-experienced mice in this study exhibited comparable MA intakes and all three groups exhibited comparable spatial recalls prior to the reversal testing.

Following the SAL/NMDA pretreatment, we failed to detect any pretreatment or sex differences in the time course for the latency to find the repositioned platform over the course of the four reversal-learning trials (Figure 18C) [Trial effect: F(3,159) = 7.700, *p* < 0.001; other *p*-values > 0.490]. However, an examination of the average latency to locate the repositioned platform yielded a significant Group effect (Figure 18C′) [F(2,58) = 7.570, *p* = 0.001; other *p*-values > 0.090]. Collapsing the data across sex (Figure 18C′), this Group effect reflected a longer average latency to locate the repositioned platform by the MA-SAL mice versus both the H2O-SAL and MA-NMDA animals (Tukey’s tests: for both comparisons, *p*-values = 0.005). Further, no difference was apparent in the average latency to locate the repositioned platform between the H2O-SAL controls and MA-NMDA mice (Tukey’s test: *p* = 0.825). Similarly, an analysis of the time course of the distance traveled across the four reversal-learning trials failed to yield a significant interaction with either the Pretreatment or Sex factors (Figure 18D) [Trial effect: F(3,159) = 9.475, *p* = 0.003; all other *p*-values > 0.356]. However, a comparison of the average distance traveled prior to locating the repositioned platform indicated group differences that were similar to those observed for the latency to reach the repositioned platform (Figure 18D′) [Pretreatment effect: F(2,58) = 7.401, *p* = 0.001; other *p*-values > 0.122; Tukey’s tests: MA-SAL vs. both H2O-SAL and MA-NMDA, *p*-values = 0.004; H2O-SAL vs. MA-NMDA mice, *p* = 0.943]. Together, these data demonstrate that systemic pretreatment with an NMDA receptor agonist reversed the MA-induced deficit in reversal learning observed in the Morris water maze, furthering the notion that the reversal-learning deficit produced by a brief history of oral MA self-administration reflects blunted NMDA receptor function.

## 3. Discussion

Below, we discuss the present findings in the context of the extant literature on the behavioral effects of repeated MA exposure and their biochemical correlates.

### 3.1. B6J Mice Consume Very Large Amounts of MA under Limited-Access Operant-Conditioning Procedures

Replicating earlier findings from our group [17], male and female B6J mice will readily respond to, and consume, unadulterated, high-concentration MA solutions (0.8–3.2 g/L) under low-demand (FR1), limited-access (1 h/day) operant-conditioning procedures (Figure 2B). While our prior high-concentration oral MA study employed a weeks-long acquisition period during which mice were first trained to respond to 0.1 g/L MA for 2–4 weeks, prior to undergoing dose–response testing for high-concentration MA intake (up to 3.2 g/L) [17], no such training procedure was employed in the present study; mice commenced their self-administration training with 0.8, 1.6, or 3.2 g/L MA, and all mice readily engaged in, and maintained, their self-administration behavior over the 7-day course of operant conditioning. To the best of our knowledge, this study is the first to demonstrate that B6J mice do not require sucrose-adulteration or -fading procedures, nor do they require low-concentration MA pre-training to entice high-concentration (>0.8 g/L) MA intake, at least under our operant-conditioning procedures. 

The females in our initial dose–response study consumed more MA from each of the three concentrations tested during the 7-day course of self-administration, relative to the males (Figure 2B). While we have reported higher oral MA intakes during the acquisition of oral MA self-administration in females versus males [17], inconsistencies exist in the literature pertaining to sex differences in MA consumption under intravenous MA self-administration by rats [49,50,51,52,53], in low-concentration (0.02–0.08 g/L) MA drinking in the home cage by mice (e.g., [19,20,21]), and in MA intake under oral operant-conditioning procedures in both mice [17,18] and rats [24,25]. Indeed, we failed to detect any sex differences in the dose–response function for MA intake across an approximately 100-fold range of MA concentrations (0.025–3.2 g/kg) in our recent oral operant-conditioning study, despite females consuming 0.1 g/L MA more than males during the acquisition phase of the experiment [17], and we detected no sex differences in the intake of the 3.2 g/L MA solution in either of our two follow-up behavioral pharmacological studies. At the present time, it is not clear why a sex difference in the MA intake was apparent in our initial dose–response experiment but not in subsequent experiments in the present study. However, when taken together with our prior results [17] and those of MA drinking published by other groups (e.g., [19,20,21]) that indicate little evidence for a sex difference in oral MA intake, the sex differences in the MA intake during our dose–response study are likely spurious.

What is clear from the results of our prior [17] and present studies is that both male and female B6J mice find MA concentrations ≥ 0.8 g/L reinforcing and will consume upwards of 30–35 mg/kg MA during a 1 h session when 1.6 or 3.2 g/L MA serve as the reinforcer (Figure 2B). This amount of oral MA is more than double the total daily dose consumed by MA-preferring DBA2/J mice or mice selectively bred for high-MA drinking when allowed 18 h access to MA in the home cage [19,20]. Of note, the MA intakes in our operant-conditioning studies were determined based on the difference between the amount of MA delivered and the MA remaining in the receptacle at the end of each operant-conditioning session, thereby providing an accurate measure of the actual amount consumed. Of note, heavy MA consumption in humans is roughly 10–13 mg/kg/day for a 170 lb person [26,28], and binge-like MA consumption can reach from 2 to 3 times these amounts [29]. Based on MA intake, our high-methamphetamine-concentration procedures (when 1.6 or 3.2 g/L MA serve as the reinforcer) meet this criterion for binge MA intake during a single 1 h session. Given that MA toxicity is prevalent in those that orally consume MA [15], in future work, it will be important to determine the blood and brain MA levels attained following our 1 h self-administration procedures. However, no mouse died during the MA self-administration or the nearly 3-week abstinence period, nor did we detect any signs of illness potentially related to organ edema [15] or kidney failure [54], which are associated with binge-like MA use in humans. 

### 3.2. A Brief History of Binge-Like MA Intake Produces Some Signs of Negative Affect in Early Withdrawal

One of the major goals of this study was to determine whether a subchronic history of high-concentration MA would be sufficient to elicit signs of negative affect in early (24 h) withdrawal, as symptoms of anxiety and depression in humans with MA use disorder are most pronounced during the first 24 h after MA cessation [4,5,55,56,57]. However, most of the MA effects observed at our 24 h withdrawal time point did not vary systemically with the amount of MA consumed. In fact, the MA-experienced mice differed only from their water-drinking controls on certain measures in the marble-burying (Figure 3A–C) and elevated plus maze (Figure 4A,E) tests, and many MA effects on our anxiety-related measures in other paradigms reflected differences between the MA-0.8 and MA-1.6 mice. Further, despite reports that MA-experienced rodents exhibit PPI deficits (e.g., [58,59]), we did not detect any MA effects on the acoustic startle or PPI in the present study (Figure 6). While an obvious explanation for our results might relate to the relatively brief self-administration history, other groups have reported robust signs of negative affect during withdrawal from either MA or amphetamine delivery procedures that resulted in lower amounts of daily drug exposure (10 mg/kg/day amphetamine via osmotic mini-pumps or MA binge-drinking procedures [19,60], or approximately 4 mg/kg/day MA via intravenous self-administration [61]). Given the large amounts of MA consumed during the course of our 7-day self-administration period (10–30 mg/kg/day), we expected more robust and consistent withdrawal signs than those observed herein. 

In humans, the time course of negative affect during early MA withdrawal is not associated with the absolute values of MA intake and can be highly variable [5]. As we examined for MA withdrawal signs at a single 24 h time point, it remains to be determined whether larger or more consistent MA effects would be observed at earlier (or even later) time points during withdrawal. Indeed, a study by Shabani et al. (2020) [19] detected increased immobility in the tail suspension test in MAHDR mice as early as 6 h following the cessation of MA drinking under their binge-drinking procedure, and this effect was also apparent at 30 h into MA withdrawal. In contrast, both the MAHDR and DBA2/J mice did not exhibit increased immobility in the forced-swim test until the 30 h withdrawal time point. Interestingly, in this same study, the DBA2/J mice failed to exhibit any effect of MA withdrawal on their behavior in the tail suspension test, which might have reflected their lower MA intake relative to that of the MAHDR mice, or the possibility that genotype may be a potential factor influencing the ability to detect the effects of MA withdrawal in certain assays of negative affect. 

In our earliest study of alcohol withdrawal in B6J mice [33], all of the paradigms employed in the present study to measure negative affect were sensitive at detecting increased negative affect during both early (24 h) and protracted (30 days) withdrawal in mice with a month-long binge-drinking history. However, subsequent reports indicated that only the light–dark shuttle-box, marble-burying, and forced-swim paradigms are consistently sensitive to the negative affective state induced in B6J mice by a shorter (2-week) alcohol binge-drinking history (e.g., [30,31]). As this study is our first to examine the negative affective consequences of oral, high-concentration MA intake, current studies in our laboratory are determining the relative sensitivity of our behavioral assays to MA withdrawal-induced negative affect by comparing mice with a 1-week versus 1-month history of high-concentration MA consumption. 

### 3.3. Withdrawal from a Brief History of Oral MA Induces a Spatial Reversal-Learning Impairment in Female Mice

Repeated MA experience induces signs of cognitive dysfunction across a number of domains in both humans (e.g., [6,7,8,9,10,11]) and laboratory rodents (e.g., [62,63]). Of relevance to the current study, MA-induced impairments in spatial learning and memory (e.g., [64,65,66,67,68,69]), as well as in reversal learning and working memory [70,71,72,73], have been reported in laboratory rodents following various MA injection regimens, particularly when MA is administered early in development. While the effects of a history of intravenous MA self-administration on recognition memory, temporal-order memory, and attentional set shifting in adult rats have been well described (cf. [62,63]), significantly less work has focused on how an MA self-administration history impacts spatial learning and memory, working memory, reference memory, or reversal learning [74,75,76,77]. Thus, another major goal of the present study was to examine the effects of our relatively brief, high-concentration, oral MA self-administration procedures on these cognitive functions.

Although a history of IV MA self-administration [78] or injection regimens intended to mimic self-administered amounts of MA (e.g., 4 or 8 mg/kg/day) [79] are reported to induce psychomotor disturbances, we detected no MA-related changes in the swimming behavior or visually cued navigation during the flag test at the start of the Morris water maze testing, conducted 2 days following the cessation of MA self-administration (Figure 7A–C). Contrary to our hypothesis, we also detected no MA effects on the rate of Morris water maze acquisition over the 4 days of training (Figure 7C–F), nor did we observe any MA–water differences during the memory probe test of the platform’s former location (Figure 7G–K). Although studies employing experimenter-injected MA to adult rodents have also reported null effects of repeated MA treatment on the Morris water maze performance (e.g., [79,80]), we predicted some sort of learning or memory impairment given the high amounts of MA consumed by the mice in the present study, and current studies are extending this investigation in mice with longer MA intake histories. However, consistent with other work indicating reversal-learning deficits following MA exposure [70,71,72,73], we did detect an impairment in reversal learning in the Morris water maze. In the dose–response study, this MA effect was female-selective (Figure 7L,M), which may reflect their relatively higher MA intake in this study. However, in our follow-up behavioral pharmacological study, during which males and females exhibited comparable MA intakes, an MA-induced reversal-learning deficit was apparent in both sexes (Figure 18). Taken together, the results of both our dose–response and behavioral pharmacological studies indicate that a relatively brief history of oral MA intake can induce a long-lasting (at least 7 days) impairment in reversal learning, at least when assayed under Morris water maze procedures. How long this deficit persists following the cessation of MA consumption, whether the severity of the deficit varies systemically as a function of the MA consumption history, and whether the deficit generalizes to other reversal-learning paradigms are all important research questions that we intend to pursue in future work. 

### 3.4. Withdrawal from a Brief History of Oral MA Induces Several Sex-Selective Changes in Protein Expression within Corticolimbic Structures

Akin to our behavioral results, an examination of the protein expression within the vPFC, dPFC, hippocampus, and amygdala also indicated relatively few water–MA differences in the expression of glutamate receptor-related proteins or their downstream effectors ERK and CamKIIα (see Table 1). Consistent with our behavioral results, most MA-related changes in protein expression did not vary systemically with the concentration of self-administered MA, nor did they align with the average MA intake of the mice. As summarized in Table 1, the MA-1.6 mice tended to exhibit the most pronounced changes in protein expression across the different brain regions, and many group differences lay between the MA-0.8 mice and MA-1.6 and/or MA-3.2 animals, rather than between the MA-experienced mice and MA-naïve controls. Also noteworthy, the majority of MA-related changes in the protein expression were sex-selective, particularly with respect to those detected within the hippocampus and amygdala (Table 1). These many sex-selective results are particularly interesting given that the males self-administered less MA than the females (Figure 1B), and we detected only a few Sex by Dose interactions with respect to behavior.

Excessive glutamate signaling is purported to be a major driver of MA-induced neurotoxicity [34,36], and both behavioral-sensitizing regimens of MA injections and intravenous MA self-administration are reported to alter both the pre- and post-synaptic aspects of glutamate signaling within the brains of primarily male rodents [37,38,39,40,41,47,48]. Despite preclinical rodent evidence that males are more sensitive than females to MA-induced neurotoxicity [81,82,83,84,85], very little work has examined the potential sex differences in MA-induced changes in the indices of glutamate transmission [52,86,87]. However, efforts to understand the biomolecular underpinnings of the sex difference in MA-induced neurotoxicity have identified sex differences in the MA-induced changes in the expression of several neuropeptides [52,88,89], immediate early genes (e.g., [90,91,92]), and kinases (incl. ERK, Akt, and glycogen synthase 3-β) [84], the time course of which also appears to be sexually dimorphic [84] (cf., [85]). In the present study, tissue was collected several weeks following the end of MA self-administration, and thus we cannot comment on the protein expression profile in early withdrawal and how it might vary with the abstinence duration. Nevertheless, our data do indicate that our relatively brief oral MA self-administration procedure is sufficient to induce several changes in the glutamate receptor expression and CaMKII activation that are apparent several weeks into withdrawal, and that the majority of these changes are sex-selective (Table 1). It will be important in future work to determine more systematically how these protein changes vary with the passage of time during MA withdrawal and with the MA-taking history, as well as the potential sex differences therein. Also, as our immunoblotting studies were designed to examine the dose–response relationships within each sex, another important facet of future work will be to directly examine the sex differences in both basal and MA-induced changes in protein expression, as they may relate to sex differences in MA-induced changes in behavior and responses to therapeutic interventions (cf., [85]).

### 3.5. Behavioral Relevance of MA-Induced Changes in mGlu1 and NMDA Receptor Expression 

Related to this latter issue, we were particularly struck by the MA-induced increase in mGlu1 expression that was apparent in the MA-1.6 mice of both sexes within the vPFC, dPFC, and amygdala and in the MA-1.6 males within the dPFC. Although negative allosteric modulators of mGlu1 are reported to exert anxiolytic effects across different rodent models (cf., [93]), including during alcohol withdrawal [94], to the best of our knowledge, the role of mGlu1 in mediating anxiety during withdrawal from amphetamines has not been examined. Herein, the potent and selective mGlu1-negative allosteric modulator JNJ 16259685, administered at a dose that reverses alcohol withdrawal-induced, anxiety-like behavior [95], reduced the marble-burying behavior of both the male and female MA-experienced mice (Figure 16). Although it remains to be determined whether mGlu1 expression is upregulated early during MA withdrawal, these results argue for an important role for mesocortical mGlu1 activity in neophobic-like behavior during early MA withdrawal. In contrast to the marble-burying test, JNJ 16259685 did not alter any behavioral measure in the MA-experienced mice when tested under the elevated plus maze or light–dark shuttle-box procedures (Figure 16). These negative findings argue that the reduction in marble burying induced by pretreatment with 5 mg/kg JNJ 16259685 likely did not reflect off-target effects on the locomotor activity. Given that we tested only a single dose of the JNJ compound in the present study, it will be important to conduct a full dose–response characterization of the effects of JNJ 16259685 on withdrawal-induced anxiety to determine conclusively whether or not its effects are selective for measures of neophobia or can be generalized across different types of anxiety. 

Spatial learning and recall in the Morris water maze, as well as spatial reference and working memory in the radial-arm maze, are highly dependent upon intact glutamate transmission within the hippocampus (cf., [95,96]). The fact that we detected only two male-selective MA–water differences in the protein expression within the hippocampus (Table 1) might account for our failure to detect robust effects of our MA self-administration procedures upon the Morris water maze and radial-arm maze performances. In contrast, many types of reversal learning are known to rely upon intact PFC function (cf., [97,98]), raising the possibility that the reduction in the obligatory GluN1 subunit of the NMDA receptor observed within the vPFC of female MA-experienced mice might relate to their impaired reversal learning in the Morris water maze. Supporting this notion, pretreatment with the non-competitive NMDA receptor antagonist MK-801 induced a reversal-learning deficit in MA-naïve mice, while pretreatment with the allosteric agonist NMDA reversed the learning deficit in MA-experienced animals, and the effects of both manipulations were sex-independent (Figure 18). The unbalanced experimental design of our NMDA study precludes conclusions regarding the MA selectivity of our observed NMDA effect, as MA-naïve mice were not included. However, the results of a prior study [99] failed to indicate any facilitatory effect of NMDA pretreatment on memory retrieval in control mice. Although we cannot discern whether the NMDA reversal of the MA-induced learning impairment is selective for MA-experienced mice, as we did not assay the effects of NMDA pretreatment on reversal learning in MA-naïve controls, NMDA pretreatment does not have an effect. Thus, although we did not detect a significant MA-induced reduction in the GluN1 expression in male mice (Table 1), the fact that the NMDA pretreatment was able to reverse the MA-induced reversal-learning impairment exhibited by both males and females suggests that our oral MA self-administration procedures reduced the NMDA receptor function in both sexes to induce this cognitive impairment. 

### 3.6. Conclusions

When trained to respond to high-concentration (0.8–3.2 g/L) MA solutions, both male and female mice will readily acquire operant conditioning for oral MA reinforcement and consume high amounts of the drug (>10 mg/kg) during a 1 h period. This relatively brief (7-day) history of oral MA is sufficient to induce some signs of anxiety-like behavior in both male and female mice, a deficit in reversal learning in female mice, and a number of sex-selective changes in the glutamate receptor expression and ERK/CaMKII activation within the vPFC, dPFC, hippocampus, and amygdala. While the precise relationships, if any, between many of the protein changes and behavioral anomalies observed during MA withdrawal and behavior require further study, the MA-induced increase in neophobia expressed during the marble-burying test depends upon intact mGlu1 signaling, while the MA-induced deficit in reversal learning reflects an MA-induced reduction in the NMDA receptor function, likely within the vPFC. 

## 4. Materials and Methods

### 4.1. Subjects

Subjects were adult (8–10 weeks of age) female (~20 g) and male (~30 g) C57BL/6J (B6J) mice (catalog no. 000664; n = 12 mice/sex/concentration), obtained from The Jackson Laboratory (Sacramento, CA, USA). Mice were housed in same-sex groups of 4 and allowed a minimum of 7 days to acclimate to a climate- and humidity-controlled colony room, under a reverse 12 h light/dark cycle (lights off at 11:00 h). Mice were identified using tail markings, and food and water were available ad libitum. All the cages were lined with sawdust bedding, with nesting materials and an igloo in accordance with vivarium protocols, and the experimental procedures complied with the Guide for the Care and Use of Laboratory Animals (2014), as approved by the Institutional Animal Care and Use Committee of the University of California, Santa Barbara (protocol number 829.4).

### 4.2. Drugs

Methamphetamine was purchased from Sigma-Aldrich (now MilliporeSigma; St. Louis, MO, USA) and was dissolved in potable water to final concentrations of 0.8, 1.6, or 3.2 g/L for oral self-administration. These MA concentrations were selected for study because we demonstrated previously that B6J mice will readily respond to MA concentrations as high as 3.2 g/L MA, orally consuming upwards of 30 mg/kg MA when this concentration serves as a reinforcer during a 1 h operant-conditioning session [17]. The NMDA receptor agonist NMDA (catalog number M3262) and the non-competitive NMDA receptor antagonist MK-801 (catalog number M107) were also purchased from MilliporeSigma and were dissolved in saline, to be administered subcutaneously at 0.1 mg/kg and 25 mg/kg, respectively. The MK-801 dose was selected based on evidence that it reduces the rodent performance in the Morris water maze, with only mild off-target locomotor effects (e.g., [100,101,102,103,104,105]), while the NMDA dose was selected based on a report that it is sufficient to reverse the amnesic effects of MK-801 in mice [99]. Finally, the highly potent and selective mGlu1-negative allosteric modulator JNJ 16259685 was purchased from Tocris (Minneapolis, MN, USA; catalog number 2333), dissolved in 0.1% DMSO, and injected subcutaneously at a dose of 5 mg/kg, based on its ability to reduce anxiety-like behavior in rats [106]. For this latter study, the 0.1% DMSO vehicle solution served as a control. 

### 4.3. Operant Conditioning for Oral MA Reinforcement

Operant conditioning for oral MA reinforcement was conducted in cohorts of 12 mice of the same sex during the dark phase of the circadian cycle. On each self-administration day, mice were relocated in their home cages to a non-colony procedural room, approximately 30 min before testing. Mice were tested in 59.69 cm × 40.64 cm operant chambers (Med Associates, St. Albans, VT, USA), housed in a sound-attenuated cabinet and equipped with two nose-poke holes (one inactive and one active/MA-reinforced) and a liquid receptacle attached to an infusion pump (Med Associates, St. Albans, VT, USA), which delivered 20 µL of the assigned MA solution (0.8, 1.6, or 3.2 g/L) into the receptacle (respectively, MA-0.8, MA-1.6, and MA-3.2). MA-naïve controls responded to potable water only (MA-0). Mice were trained to self-administer water/MA for 7 consecutive days under a fixed ratio (FR) 1 schedule of reinforcement. As in our prior study [17], each reinforcer delivery was signaled by a compound light/tone cue and 20 s time-out period. At the end of each self-administration session, mice were returned to their home cages and relocated to the colony room. At twenty-four hours following the 7th self-administration session, mice underwent a behavioral test battery to assay the effects of oral MA on the indices of negative affect and sensorimotor processing. 

### 4.4. Behaivoral Test Battery

In humans, withdrawal from binge MA self-administration sessions induces an abstinence state characterized by negative affect [3,4,5]. Thus, to determine whether our relatively brief, high-concentration MA self-administration procedures were sufficient to induce behavioral signs of MA withdrawal, mice underwent a behavioral test battery 24 h following the 7th self-administration session (see Figure 1). This behavioral test battery was similar to that employed in prior studies of alcohol withdrawal in mice (e.g., [33]) and consisted of the light–dark shuttle-box, marble-burying, novel-object reactivity, elevated plus maze, and forced-swim tests. Additionally, as evidence indicates that a history of repeated MA injections can impair the prepulse inhibition of acoustic startle (PPI) in both rats and mice [58,59,107,108], we also examined the effects of prior MA self-administration on the acoustic startle and PPI to index the sensorimotor processing. 

#### 4.4.1. Light–Dark Shuttle Box

For the light–dark shuttle-box test, mice were placed into a polycarbonate box measuring 46 cm long × 24 cm high × 22 cm wide containing 2 distinct environments for a 5 min trial. Half of the box was white and uncovered and the other half was black and covered, and these 2 environments were separated by a central divider with an opening. The animals were first placed on the dark side, and the latency to enter the light side, number of light-side entries, and total time spent in the light side of the shuttle box were recorded by experimenters who were blind to the self-administration history of the mice. Increased reluctance to venture into the light, uncovered side was interpreted as an index of photophobia/agoraphobia [109,110].

#### 4.4.2. Novel-Object Reactivity

To test the reactivity to a novel object as an index of neophobia-related anxiety, we used procedures modified from references [111,112], in which animals were placed in a novel open field (46 cm long × 42 cm wide × 40 cm high) at the center of which was placed a novel, inedible object (candlestick holder; measuring approximately 6 cm in diameter × 12 cm high). The animals’ interactions with the novel object were observed during a 2 min trial, and the number of contacts, total time spent in contact with the novel object, and fecal count were recorded by a trained observer who was blind to the self-administration history of the animals.

#### 4.4.3. Elevated Plus Maze

To increase the scientific rigor of our study and facilitate data interpretation, we also assayed mice in an elevated plus maze, which also indexes agoraphobia/photophobia. However, in contrast to the light–dark shuttle box (see Section 4.4.1), prior studies of alcohol-experienced B6 mice argue that our elevated plus maze procedures are not sensitive to the anxiogenic effects of alcohol withdrawal [30,33]. To determine whether the elevated plus maze was sensitive to the anxiogenic effects of methamphetamine, mice were placed on the center intersection of a 4-arm radial plus maze with 2 white open arms and 2 black walled arms 24 cm high. Each arm measured 123 cm long × 5 cm wide. The latency to first open-arm entry, number of open-arm entries, number of closed-arm entries, number of head dips over the edge of the open arm at the center of the maze, and total time spent in an open arm were monitored for the 2 min trial by a trained observer who was blind to the self-administration history of the mice. 

#### 4.4.4. Marble Burying

The marble-burying test was used to measure the anxiety-induced defensive burying [113]. Although some have questioned this interpretation of marble-burying behavior (e.g., [114]), ample bi-directional evidence supports the predictive validity of marble burying to index anxiety-like behavior [113,115,116,117,118,119,120,121,122], including our prior studies of anxiety-like behavior during early alcohol withdrawal (e.g., [30,31,32,33]). In our paradigm, 20 black marbles and 2 square glass pieces (2.5 cm^2^ × 1.25 cm tall) were placed in the animals’ home cage, 6 at each end. Latency to start burying the marbles was determined by a blind observer using a stopwatch, and the total number of marbles buried following a 20 min trial was recorded.

#### 4.4.5. Acoustic Startle and PPI

The apparatus and procedures employed to assay the acoustic startle and PPI were similar to those employed in earlier published work by our group (e.g., [123,124,125]). In all, six different trial types were presented: startle pulse (st110, 110 dB/40 ms); low prepulse stimulus given alone (st74, 74 dB/20 ms); high prepulse stimulus given alone (st90, 90 dB/20 ms); st74 or st90 given 100 msec before the onset of the startle pulse (pp74 and pp90, respectively); and no acoustic stimulus (i.e., only background noise was presented; st0). The St100, st0, pp74, and pp90 trials were applied 10 times, the st74 and st90 trials were applied 5 times, and all trials were given in random order. The average intertrial interval was 15 s (10–20 s), and the background noise of each chamber was 70 dB. The data for the startle amplitude were averaged across each stimulus trial type and analyzed using a Sex X Dose X Stimulus ANOVA with repeated measures on the Stimulus factor (st0, st74, st90, st110, pp74, and pp90). The percent inhibition of the 110 dB startle by the 74 and 90 dB prepulse intensities was calculated for each animal, and the data were analyzed using a Sex X Dose X Prepulse ANOVA with repeated measures on the Prepulse factor (74 vs. 90 dB). 

#### 4.4.6. Porsolt Forced-Swim Test

The Porsolt forced-swim test is a behavioral paradigm often used to evaluate the reversal of passive coping behavior by antidepressant therapies [126], and we have shown that increased swimming behavior observed during early alcohol withdrawal can be reversed by pretreatment with anxiolytic agents [123]. Therefore, we incorporated the forced-swim test as a measure of the behavioral reactivity to a physical stressor. In this assay, mice were placed into a cylindrical glass container (11 cm in diameter) filled with room-temperature water for 6 min. Using the AnyMaze tracking software (version 4.99m; Stoelting, Woodale, IL, USA), we measured the latency to the first immobile episode, the total time the animal was immobile, and the number of immobile episodes. Following completion of the test, the mice were returned to their home cages and monitored until they were dry before they were returned to the colony room. As per our IACUC protocol, the forced-swim test was always conducted last during our behavioral test battery, while the other behavioral assays were randomly administered to the mice within each cohort.

### 4.5. Morris Water Maze

The day following the behavioral test battery (see Figure 1), all mice were assayed for spatial learning and memory using the Morris water maze procedures akin to those published previously by our laboratory (e.g., [124,125,127]). The maze consisted of a stainless-steel circular tank (200 cm in diameter, 60 cm in height, and filled with room-temperature water to a depth of 40 cm), with salient intra-maze cues located on all four sides of the tank (star, sun, moon, and solid circle). Morris maze testing began with a “flag test” to ensure equivalent visual processing and swimming abilities in all mice at the outset of each experiment. For this, the clear platform was placed in the tank in the NW quadrant with a flag attached that extended 6 inches above the water. The majority of mice located the flagged platform during the 2 min period, with some mice requiring 1–2 additional 2 min training sessions. Over the course of the next 4 days, the clear platform remained in a fixed location in the SE quadrant (i.e., a quadrant distinct from that employed in the flag test). Each day, mice were trained four times a day to locate the hidden platform in the SE quadrant. During each trial, mice were randomly placed in the pool at one of the four compass points, and data pertaining to their swimming behavior were recorded digitally by a video camera mounted on the ceiling directly above the pool (ANY-Maze, Stoelting). Training sessions were 2 min in duration and mice were tested in series at each compass release point. Mice unable to locate the platform during the allotted time were guided to the platform using forceps, where they remained for 30 s. At 24 h after the last training trial, a 2 min memory probe test was performed in which the platform was removed from the pool, and the latency to arrive at the former platform location, as well as the time spent and distance traveled in the SE quadrant, were recorded. Then, a reversal-training session was conducted in which the clear platform was situated in the SW quadrant and the mice were trained to locate the platform over four 2 min sessions (one training trial for each compass point) to locate the repositioned hidden platform. 

### 4.6. Radial Water Maze

Following the Morris water maze testing (see Figure 1), the spatial working and reference memory were determined using a radial water maze with procedures described previously (e.g., [126,127]). The maze consisted of eight arms with clear, hidden escape platforms at the ends of four of the arms. The start arm was the same for all mice, and each mouse was assigned different platform locations that remained fixed throughout training, with the “baited” arms semi-randomly assigned across subjects. For each trial, a mouse had 2 min to locate a platform. If the mouse was unsuccessful at locating a platform in the allotted time, it was guided to the nearest available platform using forceps. Once a platform was found, the animal remained on it for 15 s, and it was then returned to an empty, heated holding cage for 30 s. During that time, the recently located platform was removed from the maze. The animal was then placed back into the start arm and allowed to locate another platform, and this sequence of events was repeated each day until the mouse located all four platforms. Thus, each mouse underwent four trials per day, with the working-memory system taxed increasingly with each trial. Day 1 was considered a training session because the animal had no previous experience in the maze. Days 2–10 were testing sessions, and errors were quantified for each day using the orthogonal measures of working- and reference-memory errors [128], as conducted previously by our group [124,127] and others (e.g., [129]). Working-memory correct errors were the number of first and repeat entries into any arm from which a platform had been removed during that session. Reference-memory errors were the number of first entries into any arm that never contained a platform. Working-memory incorrect errors were the number of repeat entries into an arm that never contained a platform in the past (thus, repeat entries into a reference-memory arm). In addition to these measures of working and reference memory, “chaining” behavior was also recorded. Chaining refers to the number of consecutive entries into two adjacent arms and represents an alternate strategy to maze navigation that is often exhibited by cognitively impaired subjects (e.g., [127]).

### 4.7. Immunoblotting

To determine the protein correlates of behavior, mice were decapitated 1–2 days following the last radial-arm maze session. Brains were extracted and cooled on ice, then the brains were sectioned in 1 mm thick coronal slices, and the ventral and dorsal parts of the PFC, as well as the entire amygdala and hippocampus, were dissected out, as illustrated in Figure 1. 

Immunoblotting was performed on whole-cell homogenates using procedures similar to those employed in our recent studies on glutamate receptor expression (e.g., [130,131,132,133]). The following rabbit primary antibodies were used: mGlu5 (metabotropic glutamate receptor 5; 1:1000 dilution; MilliporeSigma; AB5675); GluN1 (1:500 dilution; Cell Signaling Technology, Danvers, MA, USA; 5704S); GluA1 (1:500 dilution; Millipore; AB1504); Homer2a/b (1:1000 dilution; Synaptic Systems; 160 203); p(Thr286)-CaMKII (1:500 dilution; Cell Signaling Technology; 12716S); and ERK1/2 (1:1000 dilution; Invitrogen/ThermoFisher, Carlsbad, CA, USA; MA5-15605). The following mouse primary antibodies were also employed: mGlu1 (1:500 dilution; BD Biosciences, Franklin Lakes, NJ, USA; 610965); GluN2b (1:500 dilution; Invitrogen; MA1–2014); GluA2 (1:500 dilution; Synaptic Systems, Goettingen, Germany; 182 111); CaMKIIα (1:1000 dilution; Millipore; 05–532); p(Tyr204)ERK1/2 (1:1000 dilution; R&D systems, Minneapolis, MN, USA; AF1018); and Homer1b/c (1:1000 dilution; Santa Cruz Biotechnology, Santa Cruz, CA, USA; sc-25271). In contrast to our earlier report conducted on rat tissue [124], our selected mGlu1 antibody detected the dimer form of the receptor in mouse tissue. Thus, we present the data for both the monomer and dimer forms of this receptor herein. Calnexin expression was employed to control for protein loading and transfer using a rabbit or mouse primary anti-calnexin antibody (for rabbit primary: 1:1000 dilution; Enzo Life Sciences, Farmingdale, NY, USA; ADI-SPA-860; for mouse primary: 1:500 dilution; Enzo Life Sciences; ADI-SPA-860-D). Following primary antibody incubation, the membranes were washed with Tris-buffered saline with tween, incubated in either a goat anti-rabbit IRDye 800CW secondary antibody (1:10,000 dilution; LI-COR Biosciences, Lincoln, NE, USA; 925–3221) or goat anti-mouse IRDye 680RD secondary antibody (1:10,000 dilution; LIi-COR Biosciences; 925–68070), and imaged on an Odyssey Infrared Imaging System (Li-Cor Biosciences). Raw values for each band were measured, and they were first normalized to their corresponding calnexin signal and then to the average value of the water self-administration controls on each gel. 

Due to the large number of experimental groups in this study, immunoblotting was conducted on the tissues from males and females separately as two independent experiments. While this approach precluded any direct determination of sex differences in protein expression, it enabled a direct examination of the effects of the different MA doses within each sex of relevance in relation to the interpretation of our behavioral results.

### 4.8. Behavioral Pharmacological Studies

To determine the potential functional relevance for certain protein changes within the vPFC in medicating the effects of a brief history of oral MA on behavior, we conducted a series of small-scale behavioral pharmacological studies to target the NMDA receptor function and reversal learning in the Morris water maze, as well as the mGlu1 receptor function, in the negative affective measures influenced by MA.

#### 4.8.1. NMDA Receptor Function and Reversal Learning

The reduced expression of NMDA receptors within the mPFC is associated with impaired reversal learning (e.g., [134]). Consistent with this, the MA-3.2 mice exhibited both impaired reversal learning in the Morris water maze and reduced vPFC expression of the obligatory GluN1 NMDA receptor subunit (Figure 9D). To examine the relationship between reduced NMDA function and the MA-induced reversal-learning impairment, we first tested the effects of pretreatment with 0.1 mg/kg of the NMDA receptor antagonist MK-801 on the reversal-learning performance in MA-naïve mice. For this, a distinct cohort of experimentally naïve male and female mice were trained to locate the hidden platform under our Morris water maze procedures and were then pretreated subcutaneously with either the saline vehicle (SAL) or 0.1 mg/kg MK-801, 30 min prior to a 4-trial reversal test (see Section 4.5). Conversely, we determined the effects of stimulating NMDA receptors on the reversal-learning deficit exhibited by MA-3.2 mice by training a distinct cohort of male and female mice to self-administer 3.2 g/L for 7 days, followed by training in the Morris maze. Control mice self-administered water. Thirty minutes prior to the 4-trial reversal test, mice were injected subcutaneously with 25 mg/kg NMDA. 

#### 4.8.2. Group 1 mGlu Receptor Function and Negative Affect

Group 1 mGlu receptor antagonists exert anxiolytic effects in drug-naïve (e.g., [94,135,136]) as well as amphetamine-experienced rodents [137]; thus, we hypothesized that the altered behavior exhibited by the MA-1.6 and/or MA-3.2 mice in the elevated plus maze, light–dark shuttle-box, and marble-burying tests might reflect their elevated expression of mGlu1 in the vmPFC. To address this hypothesis, MA-3.2 g/L mice were pretreated with 5 mg/kg JNJ16259685 (JNJ) or VEH prior to testing in these paradigms.

### 4.9. Data Analyses

The behavioral data were analyzed using Sex X Dose (0, 0.8, 1.6, 3.2 g/L MA) ANOVAs, with repeated measures when appropriate. Due to the large number of experimental conditions, immunoblotting was conducted separately for male and female mice and analyzed using a one-way ANOVA along the Dose factor, followed by Tukey’s post hoc tests when appropriate. The behavioral pharmacological data were analyzed using a Sex X Pretreatment (control vs. JNJ or MK-801 or NMDA) ANOVA, with repeated measures when appropriate. For both the behavioral and immunoblotting measures, extreme outliers were first identified and excluded from the analyses using the ±3 X IQR rule. IBM SPSS Statistics software (version 27.0 for PC or Macintosh) was used for all statistical tests, and GraphPad Prism software (version 10.1.2 for PC) was used to create all graphs. Alpha was set at 0.05 for all analyses.

## 5. Conclusions

A relatively brief (7-day) history of oral MA self-administration (daily intakes > 10 mg/kg/day) was sufficient to induce some signs of negative affect in both male and female mice to impair reversal learning in female mice and to induce sometimes sex-selective changes in the expression of certain glutamate receptors and their downstream effectors within the vPFC, dPFC, hippocampus, and amygdala—although many of the MA effects did not vary systematically with the MA intake. Of relevance to the neurobiological bases and treatment of MA-induced negative affect, pretreatment with an mGlu1-negative allosteric modulator reversed the MA-induced increase in anxiety-like behavior in the marble-burying test. Supporting a key for an MA-induced downregulation in the NMDA receptor function in mediating MA-induced cognitive dysfunction, systemic pretreatment with the non-competitive NMDA antagonist MK-801 mimicked the MA-induced impairment in reversal learning, while pretreatment with NMDA reversed it. These data corroborate the results of prior studies employing experimenter-administered MA or intravenous MA self-administration procedures that indicate the MA-induced dysregulation of glutamate signaling as a potential target for the therapeutic intervention in the affective and cognitive disturbances that manifest during MA abstinence. 

## Figures and Tables

**Table 1:** Comparison of the effects of different doses of oral MA on protein expression within the vPFC, dPFC, hippocampus, and amygdala, determined approximately 3 weeks following the last MA self-administration session. Bolded results highlight significant differences between MA-naïve (0 mg/L) controls and at least one MA-experienced group. Sex-selective results are also highlighted in red and blue for female- and male-selective effects, respectively. Graphical depictions of these group differences are presented in Figure 9, Figure 10, Figure 11, Figure 12, Figure 13, Figure 14, Figure 15 and Figure 16.

Brain Region	vPFC	dPFC	Hippocampus	Amygdala
GluA1	1.6 > 3.2 (F)			1.6 > 0.8 & 3.2 (M)
GluA2	0.8 > 3.2 (F)			
GluA2:GluA1 ratio				**0, 0.8 & 3.2 < 1.6 (M)**
GluN1	**0 & 0.8 > 3.2 (F)**		0.8 < 1.6 (M)	0.8 < 1.6 & 3.2 (M)
GluN2A			0.8 < 1.6 (M)	**0 & 0.8 < 1.6 (F)** **0, 0.8 & 3.2 < 1.6 (M)**
GluN2B				**0, 0.8 & 3.2 < 1.6 (M)**
mGlu1 monomer	**0 < 1.6**	**0 < 1.6 (F)** **0 & 0.8 < 1.6 (M)**	**0 & 0.8 < 1.6 (M)**	**0 & 0.8 < 1.6 (F)** **0 & 0.8 < 3.2 (M)** 3.2 > 0.8 (M)
mGlu1 dimer				**0 & 0.8 < 1.6 (F)**
mGlu5 monomer	**0 < 1.6 & 3.2 (M)**	**0 < 1.6 & 3.2 (F)**		
mGlu5 dimer	**0 < 3.2** 0.8 < 3.2 (M)			0.8 < 1.6 (M)
Homer1b/c				0.8 < 3.2 (M)
Homer2a/b			0.8 < 1.6 (M)	0.8 < 1.6 (M)
ERK			0.8 < 1.6 & 3.2 (M)	
p-ERK	0.8 > 1.6 & 3.2			0.8 < 1.6 & 3.2 (M)
p-ERK:ERK ratio				
CaMKII				0.8 < 1.6 & 3.2 (M)
p-CaMKII	1.6 < 3.2 (F) **0, 0.8 & 1.6 < 3.2 (M)**		1.6 < 3.2 (F) **0, 0.8 & 1.6 < 3.2 (M)**	
p-CaMKII:CaMKII ratio	**0 & 1.6 < 3.2**		**0 & 1.6 < 3.2**	

## Data Availability

The data presented in this study are available upon request from the corresponding author.
